# Nanomaterial Fabrication through the Modification of Sol–Gel Derived Coatings

**DOI:** 10.3390/nano11010181

**Published:** 2021-01-13

**Authors:** Wai Kian Tan, Hiroyuki Muto, Go Kawamura, Zainovia Lockman, Atsunori Matsuda

**Affiliations:** 1 Institute of Liberal Arts and Sciences, Toyohashi University of Technology, Toyohashi, Aichi 441-8580, Japan; muto@ee.tut.ac.jp; 2 Department of Electrical & Electronic Information Engineering, Toyohashi University of Technology, Toyohashi, Aichi 441-8580, Japan; kawamura.go.km@tut.jp; 3 School of Materials and Mineral Resources, Engineering Campus, Universiti Sains Malaysia, Nibong Tebal 14300, Penang, Malaysia; zainovia@usm.my

**Keywords:** sol–gel, coating, hot-water treatment, oxide materials, composite materials, nano-coating, thin film, micropatterning, optical properties

## Abstract

In materials processing, the sol–gel method is one of the techniques that has enabled large-scale production at low cost in the past few decades. The versatility of the method has been proven as the fabrication of various materials ranging from metallic, inorganic, organic, and hybrid has been reported. In this review, a brief introduction of the sol–gel technique is provided and followed by a discussion of the significance of this method for materials processing and development leading to the creation of novel materials through sol–gel derived coatings. The controlled modification of sol–gel derived coatings and their respective applications are also described. Finally, current development and the outlook of the sol–gel method for the design and fabrication of nanomaterials in various fields are described. The emphasis is on the significant potential of the sol–gel method for the development of new, emerging technologies.

## 1. Introduction

### 1.1. The Sol–Gel Method

Since its discovery in the 1970s, the sol–gel method has been rapidly developed and used for materials engineering in various fields [1,2]. The prospects of the sol–gel process were described by Ulrich [3] in 1988 and by Hench in 1990 [4], and its potential has been increasing significantly until now. The sol–gel route is a well-known wet chemical method that allows low-temperature material synthesis with excellent composition and homogeneity control. The sol–gel method also offers simple fabrication with good potential for large-scale fabrication, most importantly at a minimal cost [5,6,7].

Generally, inorganic or metal organic precursor compounds are dissolved in an alcohol solvent, followed by hydrolysis with water and a condensation process to form dispersed fine particles or polymers known as sol. The fundamental sol–gel chemistry has been comprehensively covered by other reviews [8,9]. In brief, the formation of interconnections between sols creates an inorganic polymer network, known as gel, which still contains remnant water and solvents. Formation or coating is carried out in the transition window from sol to gel prior to removal of the remnant water and solvents, forming a dry gel. Heat treatment of the gel then enables the formation of dense final products [10].

Regarding the sol–gel precursor materials, Kessler has written a comprehensive chapter on sol–gel synthesis through homometallic and heterometallic precursors [11]. In the sol–gel method, the most commonly used metalorganic compounds are metal alkoxides (M(OR)_z_, where R and z represents an alkyl group (C_x_H_2x+1_) and a valence number of M, respectively) [11,12]. By chemically modifying the alkoxides with ligand complexes, the hydrolysis and condensation rates can be moderated [13,14].

With rapid technological advancement, material engineering via the sol–gel route has also advanced significantly. For example, in a recent interesting development, the fabrication of high-performance ultrafine alumina fiber using the sol–gel method via centrifugal spinning, electrospinning, and electro-blown spinning are reported [15]. Using advanced precision equipment and by adjusting the sol–gel process parameters, fabrication of ultrafine alumina fibers with excellent controllability in terms of diameter, composition, component, and structure is reported.

### 1.2. Outline of This Review

In previous reported reviews, Aurobind et al., discussed the process steps used for silica-based gels and xerogels fabrication, followed by doping process that was carried out to achieve improved stability and desired property for pH sensing applications [8]. In addition to excellent reproducibility, the sensors obtained using this method exhibited improved chemical and mechanical stability. Meanwhile, Nistico et al., presented the formation of inorganic porous coatings via sol–gel route, focusing on sol–gel chemistry, templating method (soft vs. hard) and spin-coating deposition [9].

In this review, after a brief description of the sol–gel method and the formation of sol–gel derived coatings, the modification of sol–gel derived coatings via various methods will be discussed. This includes a comprehensive survey of oxide materials and their composites fabrication for different applications. Finally, recent materials development using the sol–gel method in the latest emerging technologies is also mentioned prior to the summary of this review.

## 2. Formation of Sol–Gel Derived Coatings

**Figure 1:**
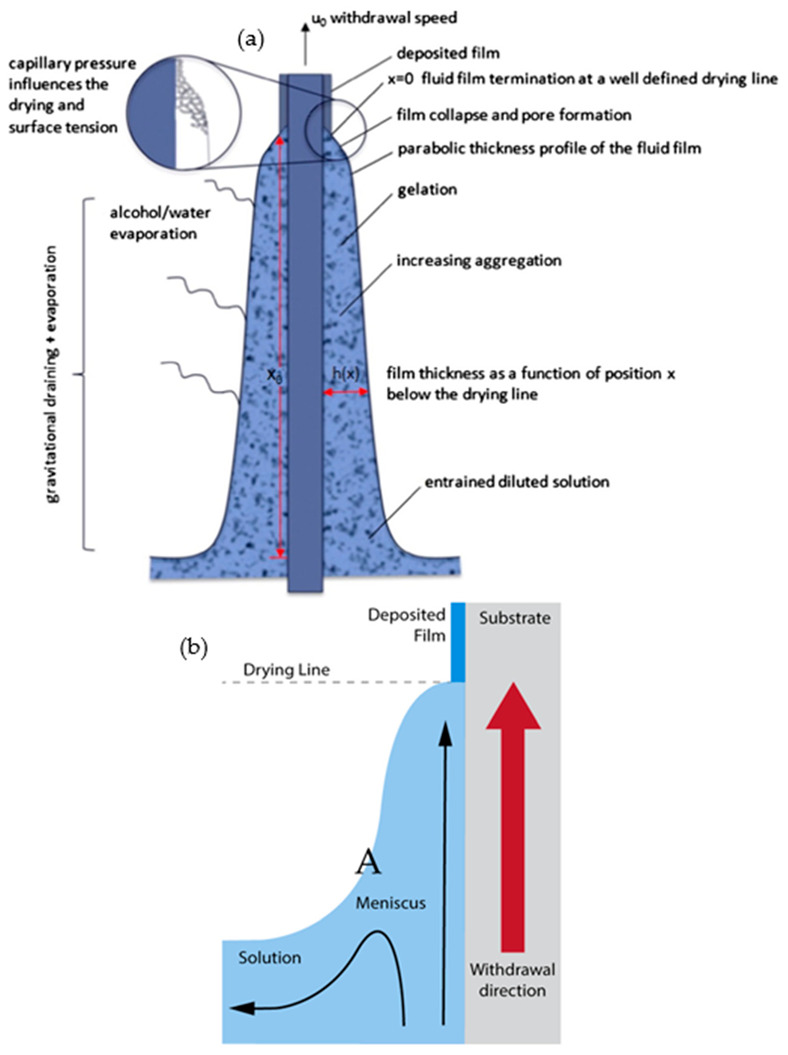
(**a**) Schematic of the steady-state dip coating process and (**b**) streamline directions during the dip coating. Reprinted with permission from [17], copyright (2016) Elsevier.

The sol–gel method is regarded as a promising technique for thin film fabrication and is commonly used in glass and inorganic film formation. Simple and effective coating techniques that are generally used include dip coating, spin coating, spray coating, and roll coating. The fundamental process for these coatings involves the entrainment of liquid causing a two-way free surface split in a viscous boundary layer. The outer layer would be pulled back into the sol bath. The following evaporation would then generate a wedge that acts as the termination point forming a distinct drying line (point x = 0 in Figure 1a). When a balanced of flux movement (depending on the coating methods) and evaporation is achieved above the stagnation point (point A in Figure 1b), a stable film in term of shape and position, is obtained. The continuous evaporation of the coating would further concentrate the inorganic substance. This would result in aggregation, gelation, and upon a complete drying process, a dry gel (also known as xerogel) is formed. Brinker et al., reported on this phenomenon in the early 1990s that elucidated the underlying chemistry and physics of sol–gel thin film deposition by dip coating [16]. Their findings enable a clear understanding of the mechanism allowing further development in process control. For example, in dip coating and spin coating, the thickness of a coating can be adjusted by changing the withdrawal or spinning speed, respectively. Nevertheless, the property of the sol used also strongly influences the formation of the coating profile.

Depending on the type of materials used in the formation of the sol–gel derived coatings, unique properties can be generated and controlled, such as those for electrowetting, oriented seed layer formation, and templates for nano- and microstructural engineering. By controlling the formation parameters as well as template removal, novel architectonic nanostructures can be formed for various applications using the sol–gel route. These have led to a vast number of reported studies on the modification of sol–gel derived films and coatings. Owing to the large volume of literature, it is impossible to cover all the topics related to sol–gel coatings and their modification. However, the following sections will highlight part of the reported studies with interesting findings used in the past decades and recent years.

In Section 3, the formation and modification techniques of sol–gel derived coatings are described, covering sol–gel films patterned by electrophoretic and photo-irradiation as well as low temperature crystallization of sol–gel derived coatings via hot-water treatment. As for hot-water treatment of sol–gel derived coatings, several different oxide materials’ formation since this method’s discovery, until recent years, are mentioned. Whereas, in Section 4, the application of sol–gel technology in recent emerging applications such as fuel cells, batteries, super-wettability control, extraction technology, nanocomposites formation, and periodic templating as well as hierarchical nanostructures formation, are described. The ease of sol–gel modification via the abovementioned techniques generating desired material properties, further emphasizes the vast potential of this method for the development of advanced functional materials.

## 3. Formation and Modification of Sol–Gel Derived Coatings

### 3.1. Electrophoretic Patterning of Sol–Gel Films

Electrophoretic deposition is a well-known, versatile technique for the deposition of thin or thick films on conductive substrates under an electric field [18,19,20]. The fast deposition rate that enables thick-film formation is one of the advantages of this technique. By controlling the applied voltage, deposition time, and suspension concentration, the thickness of the deposited film can be controlled [2]. For electrophoretic deposition, a stable suspension with uniform particle distribution is vital for obtaining an excellent deposited film. Therefore, in electrophoretic sol–gel deposition, sol–gel derived particles are used. This process can be applied for various applications.

**Figure 2:**
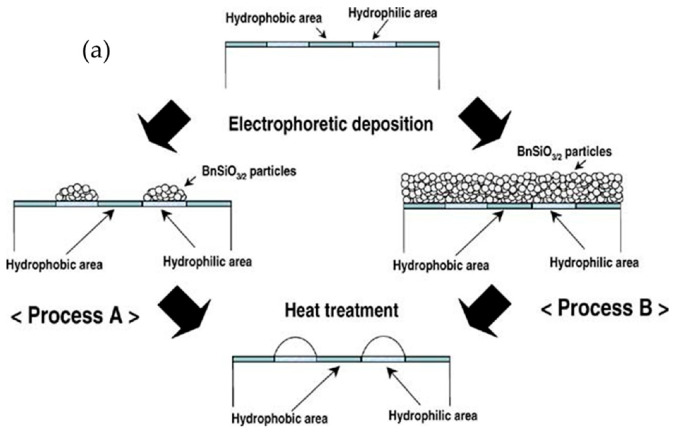

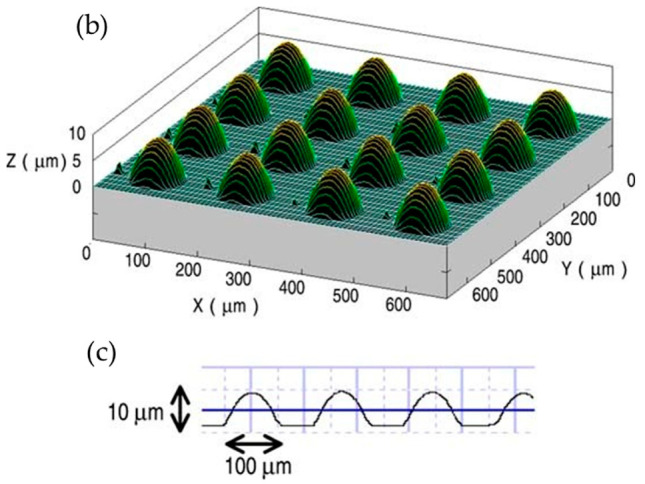
(**a**) Schematic for the preparation of BnSiO_3/2_ micropatterns by the electrophoretic sol–gel deposition process using a hydrophobic–hydrophilic-patterned surface. (**b**) Three-dimensional surface profile and (**c**) cross-sectional profile of convex-shaped BnSiO_3/2_ micropatterns prepared by electrophoretic sol–gel deposition using a hydrophobic–hydrophilic-patterned surface and heat treatment at 200 °C for 30 min. Reprinted with permission from [24], copyright (2007) Springer.

Through micropatterning, thick transparent sol–gel films can be applied as wavelength guides and micro-optical components. Using the electrophoretic sol–gel deposition of benzyl-silsesquioxane (BnSiO_3/2_) molten liquid between a hydrophobic fluoroalkylsilane surface and a hydrophilic silica surface, Takahashi et al., demonstrated the feasibility of micropatterning transparent BnSiO_3/2_ thick films, as shown in the schematic of Figure 2a. Using indium tin oxide glass as the substrate, hydrophobic–hydrophilic patterns were prepared using the sol–gel method prior to the electrophoretic deposition of BnSiO_3/2_. Then, heat treatment above the glass transition temperature led to the migration of BnSiO_3/2_ liquids from the hydrophobic areas to the hydrophilic areas [21,22,23,24]. The three-dimensional surface and cross-sectional profiles of convex-shaped BnSiO_3/2_ micropatterns are shown in Figure 2b,c, respectively. 

### 3.2. Photo-Irradiation of Sol–Gel Coatings

In the micropatterning of sol–gel derived films, laser photo-irradiation is one of the methods used to densify a local region of the film prior to etching to remove the unwanted regions. In the early days, the feasibility of photo-assisted micropatterning was demonstrated by Tohge et al. [25]. By modifying the metal alkoxides with β-diketones, the micropatterning of oxide gel film was demonstrated using UV irradiation. Owing to the π–π* transition of β-diketone, the complexes formed exhibited adsorption in the UV region. After UV irradiation through a photomask, the films were then leached using aqueous nitric acid solution or ethanol before heat treatment to remove the residual organic compounds.

Formation and patterning of conductive tin oxide films by UV irradiation was also demonstrated by Tadanaga et al., using SnCl_2_ and acetylacetone as precursors. After leaching of the photo-irradiated films, leaching in NaOH or NH_4_OH solution was carried out before heat treatment. The dimensions of the obtained pattern were 3–50 μm in width and approximately 0.1 μm in thickness, with a pitch of 2–20 μm. The resistivity reported after heat treatment at 500 °C was approximately 1 × 10^−2^ Ω cm [26].

**Figure 3:**
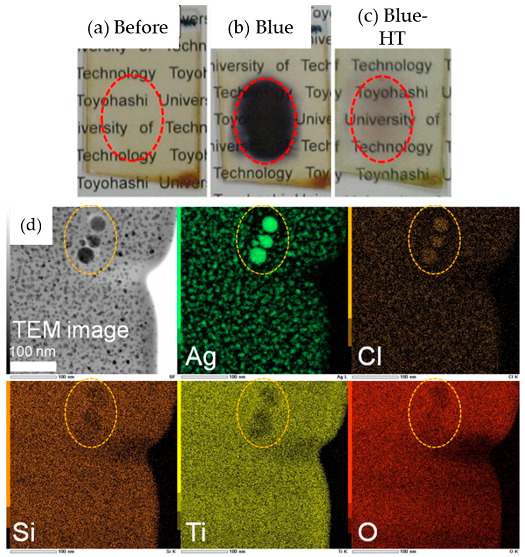
(**a**–**c**)Photographic images of a 30Ag15Cl:40GPSiO_3/2_-60TiO_2_ (GP: Glycidoxypropyl, mol%) film before and after blue laser irradiation and subsequent heat treatment. (**d**) Cross-sectional TEM image and corresponding EDX mapping of a 30Ag15Cl:40GPSiO_3/2_-60TiO_2_ film after blue laser irradiation. The circles indicate the same region in all the sections. Reprinted with permission from [28], copyright (2011) Elsevier.

In the development of holographic materials using the photo-irradiation of the sol–gel derived films, Kawamura et al., demonstrated that blue laser irradiation of AgBr-doped organo-silsesquioxane–titania (RSiO_3/2_-TiO_2_) films (thickness of approximately 5 μm) could convert the AgBr crystals into very small Ag nanoparticles (AP), which led to enhanced absorption of the film in the visible region. The two-beam interference exposure of the blue laser was considered to be the origin of the diffraction efficiency variation [27]. In a subsequent study, they further demonstrated a reversible conversion using sol–gel derived film by adding TiO_2_ to the organic–inorganic nanocomposite films [28]. Interestingly, reversible recording of the films was achieved by blue laser irradiation and subsequent heat treatment. The key step lies within the partial conversion of AgCl nanocrystals in the sol–gel films to Ag and its conversion back to AgCl by heat treatment, leading to reversibility in absorbance without altering the morphology of the NP dispersion. The presence of TiO_2_ increased the film density and played an important role in suppressing the movement of Cl and the leaching of Cl from the film. This was required for recombination with the Ag NPs to reform AgCl during heat treatment, enabling the film to return to its initial state, as shown in Figure 3.

### 3.3. Low-Temperature Crystallization of Sol–Gel Derived Coatings by Hot-Water Treatment

Despite the many advantages of sol–gel derived coatings, one of the drawbacks is that the initial coating formed is in an amorphous state; therefore, heat treatment (in the region of 300 °C to 800 °C) is indispensable for promoting crystallization of the films [29]. This requirement is a setback for the usage of sol–gel derived coatings on polymeric substrates and other materials with low thermal stability. The feasibility of obtaining crystallized sol–gel derived coatings at low-temperatures will allow its application in the development of flexible devices that use conductive polymeric substrates, as well as reduce energy consumption for the fabrication of affordable devices. In the early 2000s, Matsuda and colleagues reported on the low-temperature crystallization of sol–gel derived coatings using a hot-water treatment (HWT). The crystallization and formation mechanism involve the (i) hydrolysis of sol–gel layer and dissolution of organic component, (ii) migration of hydrolyzed inorganic species from within the sol–gel derived film to the coating’s surface and finally, (iii) nucleation and growth of the nanocrystals on the residual coating [29,30,31,32]. This shows that the HWT mechanism involves a dissolution of the sol–gel derived film and followed by precipitation of inorganic species onto the residual layer via nucleation and growth. Since then, various types of hot-water-treated, sol–gel derived coatings, and their potential applications have been reported [30,32,33,34,35,36]. The following section describes the HWT of sol–gel derived coatings and the parameters used for the controlled formation and modification of various sol–gel coatings for different applications.

#### 3.3.1. HWT of SiO_2_–TiO_2_ Sol–Gel Derived Coatings

Titanium dioxide (TiO_2_) is a well-known photocatalyst with many exceptional properties. One way to obtain TiO_2_ nanocomposites, as well as porous films with high surface areas, is to use the sol–gel method. The presence of TiO_2_ in anatase form is the most beneficial for photocatalysis applications, but the required heat treatment leads to the formation of a rather opaque film. In a novel low-temperature fabrication of anatase TiO_2_, Matsuda et al., demonstrated the feasibility of forming transparent anatase nanocomposite films by hot-water treating SiO_2_–TiO_2_ gel films [30]. They also reported that the addition of poly(ethylene glycol) (PEG) into the SiO_2_–TiO_2_ gel films led to the formation of TiO_2_ anatase crystals within the entire film, compared with the formation of the crystals only at the surface when PEG was not added [37]. The mechanism involved during the HWT was the following: (i) hydrolysis of Si–O–Ti bonds and dissolution of the SiO_2_ component, (ii) migration of hydrolyzed titania species from the interior to the surface of the coating, and (iii) nucleation and growth of titania nanocrystals in the residual coating.

**Figure 4:**
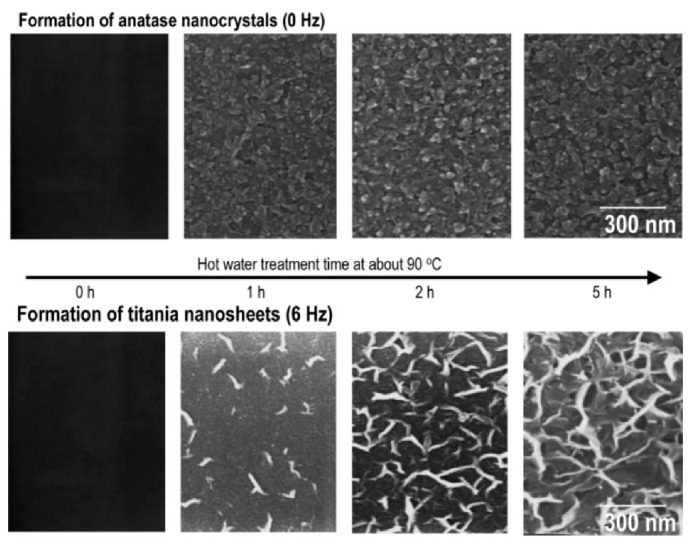
SEM images of the formation of granular anatase TiO_2_ precipitates without vibration, and sheet-like precipitates generated with mechanical vibration at 6 Hz on 75SiO_2_·25TiO_2_ (mol%) coatings during hot-water treatment (HWT) at 90 °C. Reprinted with permission from [31], copyright (2005) ACS.

In a different study, the effects of vibration during HWT on the formation of TiO_2_ crystallites were reported. During HWT with vibration of the 75SiO_2_·25TiO_2_ sol–gel coatings, hydrated TiO_2_ with a lepidocrocite-like layered structure was obtained, as shown in Figure 4 [31]. Interestingly, the precipitated sheet-like TiO_2_ exhibited good photocatalytic properties with excellent wettability and antifogging properties after HWT of the coatings at a low-temperature of 90 °C under mechanical vibration at 6 Hz.

By applying a longer HWT to the SiO_2_–TiO_2_ sol–gel coatings at a relatively lower temperature, the precipitation of smaller TiO_2_ anatase crystals with a large specific surface area was reported to promote higher photocatalytic activity. HWT at temperatures as low as 30 °C was conducted for 65 h, and Matsuda et al., demonstrated that the sol–gel coatings that were preheated (60 °C, 1 h) and followed by HWT at 55 °C for 15 h exhibited higher photocatalytic properties in methylene blue degradation compared with those obtained at higher temperatures [38]. In a different study, it was also demonstrated that HWT of cetyltrimethylammonium bromide (CTAB) incorporated SiO_2_–TiO_2_ sol–gel coatings led to the formation of TiO_2_ anatase crystals over the entire film compared with surface formation only without the CTAB addition [39]. The presence of CTAB generated a template-forming mesoporous structure during the HWT when the CTAB was removed and replaced by the TiO_2_ nanocrystals. The films obtained after the HWT exhibited good photocatalytic activity in the photogeneration of I_2_, owing to the large porous surface structure as well as superhydrophilicity. 

**Figure 5:**
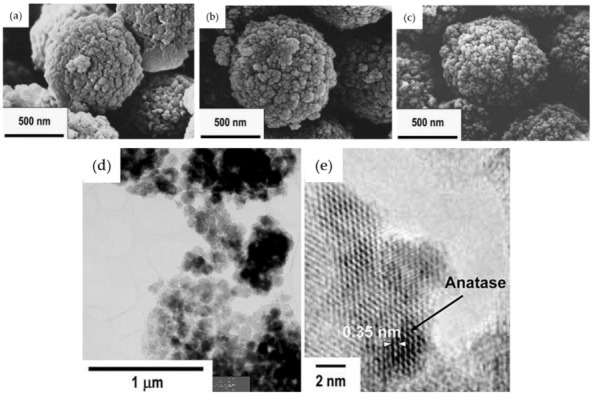
FE-SEM images of the SiO_2_–TiO_2_ microparticles, (**a**) before HWT, (**b**) after HWT for 1 h, and (**c**) 4 h. The TEM and high-resolution TEM images of the SiO_2_–TiO_2_ microparticles obtained after HWT for 4 h are shown in (**d**,**e**), respectively. Reprinted with permission from [40], copyright (2006) Springer.

Using the SiO_2_–TiO_2_ microparticles obtained after the HWT, their group further demonstrated the possibility of achieving controlled film deposition using electrophoretic deposition [40]. Field emission scanning electron microscope (FESEM) images of the 75SiO_2_·25TiO_2_ (mol%) microparticles obtained before and after the HWT for 1 and 4 h are shown in Figure 5a–c. Although the shape and size of the microparticles remained the same after the HWT, their surface texture appeared to be aggregated, owing to the precipitation of TiO_2_ anatase crystals. This was proven by the transmission electron microscope (TEM) images shown in Figure 5d,e indicating the formation of 50-nm anatase crystals with a lattice fringe of 0.35 nm, which corresponded to TiO_2_ anatase (101) [40]. Using the microparticles, a thick film of approximately 10 µm was obtained using electrophoretic deposition, which exhibited an improved photocatalytic reaction in KI aqueous solution compared with dip-coated film.

**Figure 6:**
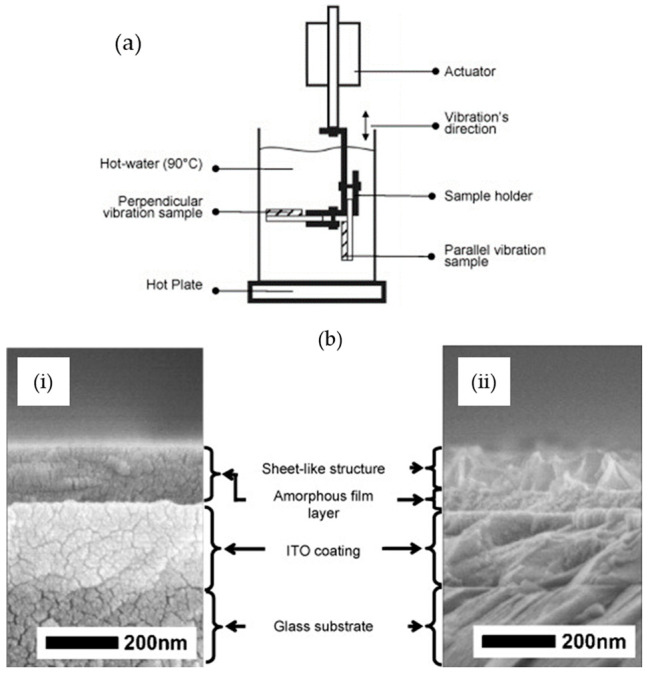

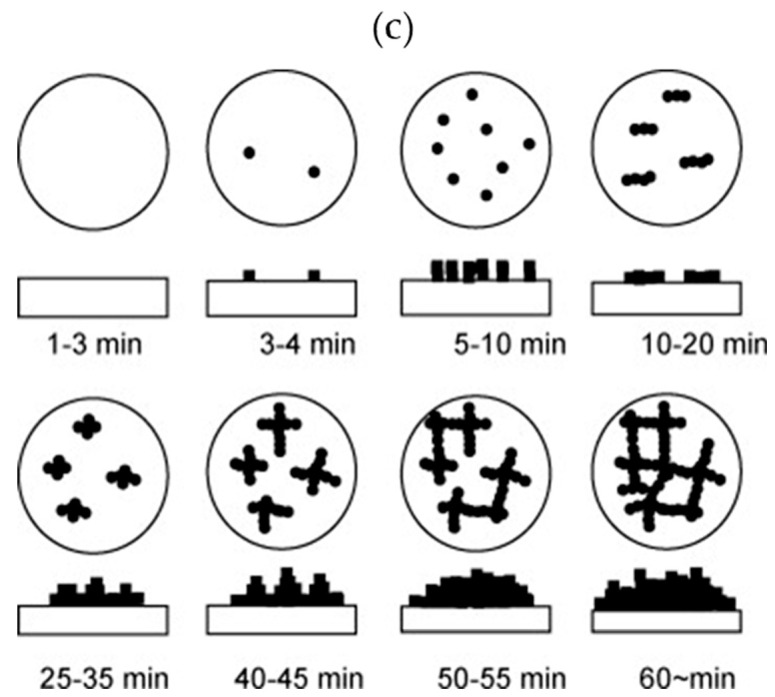
(**a**) Schematic of vibration HWT experimental method. (**b**) Field emission scanning electron microscope (FESEM) image characterization of 87.5SiO_2_·12.5TiO_2_ (mol%), (**i**) before treatment and (**ii**) after treated by hot-water parallel vibration at 90 °C for 3 h. (**c**) Formation mechanism of titania nanosheet crystallites on silica–titania gel films by vibration HWT. Reprinted with permission from [32], copyright (2009) Elsevier.

Despite numerous reports on the formation of TiO_2_ crystals by the HWT of SiO_2_–TiO_2_ sol–gel coatings, the detailed mechanism was not reported until the systematic investigation by Prastomo et al. [32,41]. In their study, they also concurrently applied either perpendicular or parallel vibration treatment, as shown in the schematic in Figure 6a, to obtain a controlled formation of TiO_2_. They discovered that during the migration and reprecipitation process, the TiO_2_ nanocrystals tended to precipitate in the microcracks of the film. The obtained cross-sectional FESEM morphologies and the mechanism are shown in Figure 6 b,c, respectively. After HWT with parallel vibration at 90 °C for 3 h, the resulting film consisted of an amorphous layer and a hydrated, sheet-like TiO_2_ nanocrystal layer. The schematic of the mechanism, shown in Figure 6c, indicates the formation sequence as follows: (i) formation of spherical precipitates, (ii) formation of elongated precipitates, (iii) interconnection of elongated precipitates with one other, and (iv) formation of the sheet-like structure from the growth and combination of the elongated precipitates. Concurrent parallel vibrational treatment during HWT was also mentioned as being preferable for the formation of sheet-like TiO_2_ [32].

In a separate study, the effects of an electric field during HWT were also further investigated. Owing to the concentration gradient at the surface, the control of the precipitation process can be controlled by either electric field application or mechanical vibration agitation [41]. When an electric field of less than 10 V cm^−1^ was applied to the negative electrode of the substrates during HWT at 90 °C of 75SiO_2_·25TiO_2_ (mol%) coatings, Matsuda et al., reported the formation of ramiform TiO_2_ anatase. The resultant films remained transparent after HWT with an applied voltage below 5 V cm^−1^. However, when the voltage was increased from 5 to 10 V cm^−1^, the ramiform shape of the TiO_2_ became more significant and a gradual transition from a light colored Ti^4+^ to a darker colored Ti^3+^ occurred because of a reduction process that lowered the transmittance properties. Interestingly, the colored coatings turned transparent upon heat-treatment in air at temperatures higher than 400 °C. It was also reported that the transparent coatings with ramiform-structured TiO_2_ after HWT at 90 °C for 5 h under 5 V cm^−1^ exhibited excellent water wettability with a contact angle of less than 5° [42]. 

#### 3.3.2. HWT of Zirconium Oxide Sol–Gel Derived Coatings

**Figure 7:**
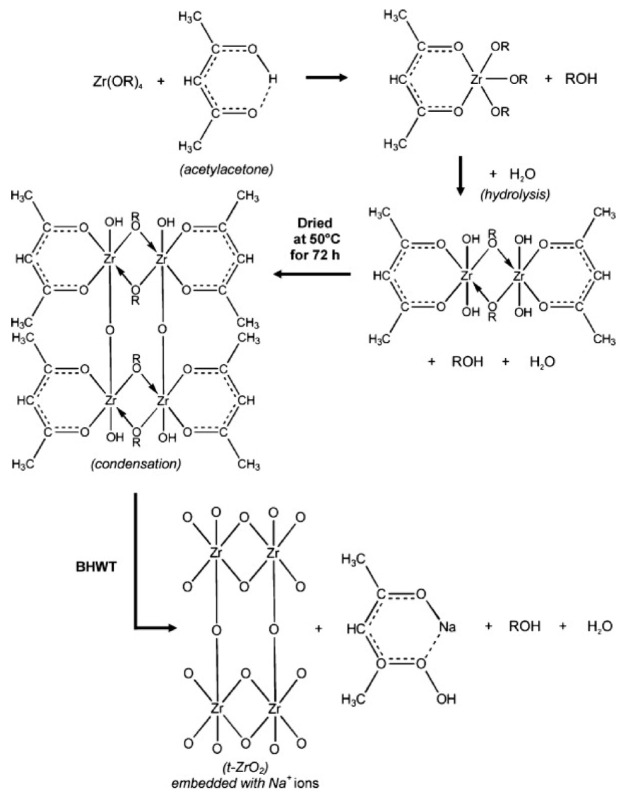
Formation mechanism and crystallization of high surface area ZrO_2_ sol–gel derived NPs by basic HWT. Reprinted with permission from [34], copyright (2010) Elsevier.

Besides TiO_2_, it has also been demonstrated that HWT can be used for the crystallization of sol–gel derived zirconium oxide (ZrO_2_) powder, but under basic conditions [34]. This is due to the low solubility of ZrO_2_ in water at the neutral condition (pH 7). Using the HWT (90 °C) of ZrO_2_ gel powders at pH 14 with the addition of NaOH, the formation of tetragonal-phase ZrO_2_ was obtained. The concurrent effective removal of organic compounds from the sol–gel derived ZrO_2_ NPs also occurred. The precursor used in their study was zirconium n-butoxide (Zr(n-OBu)_4_) and acetylacetone was applied as a chelating agent [34]. A hydrolysis reaction was induced using ion-exchanged water with nitric acid as a catalyst. They reported that basic HWT is crucial for the preparation of fine ZrO_2_ crystals with a high surface area. The specific surface areas obtained for the amorphous as-obtained gel after drying and the tetragonal ZrO_2_ (crystal size of 18 nm) after basic HWT for 24 h were 17 and 275 m^2^/g, respectively. Based on the reaction that occurred during the basic HWT, Prastomo et al., proposed the formation mechanism, as shown in Figure 7. First, there is a reaction between the dissolved zirconia alkoxide in ethanol and the acetylacetone-generated, chelated zirconia–acetylacetone complex. During the hydrolysis process, an interconnected network structure is formed, which is then condensed upon drying. When the chelated ZrO_2_–acetylacetone complex is hot-water treated in a basic NaOH solution, porous and organic free ZrO_2_ is formed with the dissolution of acetylacetone, from the complex into the solution, as sodium acetylacetonate. At a relatively high HWT temperature (90 °C), a higher reaction rate is promoted, leading to an increased collision rate of the particles. The unpaired bonds that are generated after the organic compound removal then provide sites for further strengthening of the ZrO_2_ interconnected network. This enables the formation of a basic hot-water treated gel structure with a high surface area. Besides that, the simultaneous incorporation of Na ions into the ZrO_2_ structure plays an important role in the stabilization of ZrO_2_ crystals, favoring the formation of tetragonal-phase ZrO_2_ [34].

**Figure 8:**
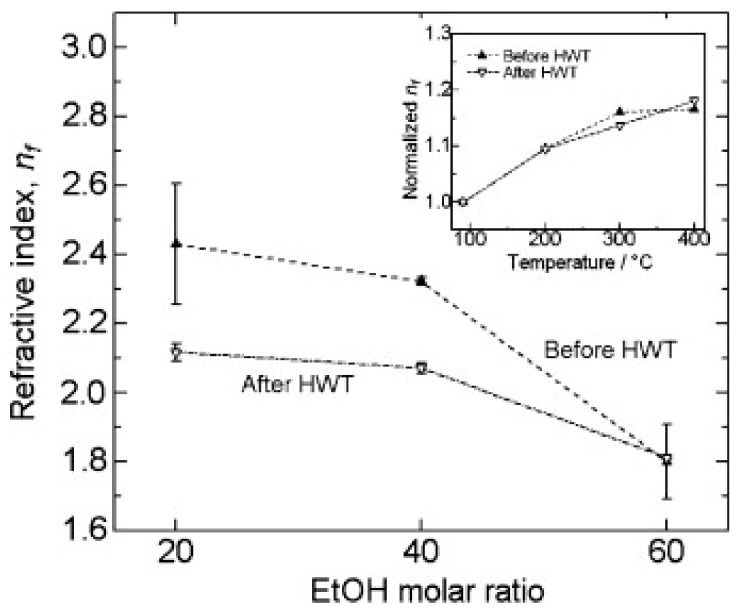
Refractive index of ZrO_2_ thin film as a function of the molar ratio of ethanol/Zr[O(CH_2_)_3_CH_3_]_4_ before and after HWT (annealed at 400 °C). Inset is the normalized refractive index of ZrO_2_ film before and after HWT, which was annealed at various temperatures (molar ratio of ethanol: Zr[O(CH_2_)_3_CH_3_]_4_ = 60:1.25). Reprinted with permission from [7], copyright (2012) Elsevier.

In a subsequent development, Soo et al., demonstrated the influence of the ethanol molar ratio and annealing temperature of ZrO_2_ sol, prepared using Zr[O(CH_2_)_3_CH_3_]_4_ alkoxides in the presence of ethanol (EtOH), nitric acid, and acetylacetone. They also elaborated on the effect of HWT on the film thickness, refractive index, and crystal size in the generated ZrO_2_ films [7]. In addition, they obtained films with different thicknesses using spin coating, by adjusting the molar ratio of the ethanol. Furthermore, they discovered that HWT on the ZrO_2_ sol–gel derived films led to a film thickness reduction, whereas the crystallinity of the oxide films improved with HWT prior to heat treatment [7]. The obtained ZrO_2_ films exhibited high transmittance properties (higher than 80%) for the annealed samples (both before and after HWT). Moreover, from the analysis that was carried out, they reported that the refractive index could be controlled by adjusting the molar ratio of the ethanol used, as well as the annealing temperature. The refractive index obtained was in the region of 1.6 to 2.4, which was influenced by the microstructure and packing density of the generated films. As shown in Figure 8, the refractive index decreased when the higher molar ratio of ethanol to Zr[O(CH_2_)_3_CH_3_]_4_ was used. This was caused by the reduction of the ZrO_2_ film thickness, which affected the interaction of light with the ZrO_2_ films, increasing the transmittance while reducing the refractive index [43]. Coarsening of the films with higher porosity after HWT also caused the refraction index to decrease when molar ratios of ethanol of 20 and 40 to Zr[O(CH_2_)_3_CH_3_]_4_ were used.

#### 3.3.3. HWT of Al_2_O_3_ Sol–Gel Derived Coatings

With the increasing demand for antireflective coatings for optical devices, the development of antireflective-glass-based optical materials has also increased [44,45]. The formation of flowerlike alumina pseudoboehmite nanocrystals has been reported, and the density gradient from the surface of the film to the interface between the film and substrate generated a “moth’s-eye” effect, which can reduce reflection from the surface. A study on the low-temperature formation of alumina antireflective coating by the HWT of the obtained sol–gel film was investigated by Tadanaga et al., using aluminum tri-sec-butoxide and ethylacetate on soda lime glass substrates. After the HWT, pseudoboehmite nanocrystals formed on the surface. With the dense gradient generated from the surface to the coating-substrate interface, there was a refractive index gradient that led to antireflective property of lower than 0.5% in the wavelength region of 360 to 620 nm [46]. Given the advantage of low-temperature crystallization using HWT, this technique is applicable for polymeric substrates with low thermal stability.

They also demonstrated that antireflective alumina films could be formed on various polymer substrates, such as poly(ethylene terephthalate) (PET) and polycarbonate (PC) [47]. They further reported on the usage of poly (methyl methacrylate) (PMMA) substrates for the formation of antireflective flowerlike pseudoboehmite nanocrystals using the above-mentioned precursor. They achieved a reflectance lower than 0.8% in the visible light region and a moth’s-eye structure phenomenon of the coatings on PMMA caused by to an extremely low incident angle dependence of the antireflective properties [48]. These findings confirm that the HWT of sol–gel derived coatings can be applied for the formation of antireflective coatings on polymeric substrates.

#### 3.3.4. HWT of ZnO Sol–Gel Derived Coatings

**Figure 9:**
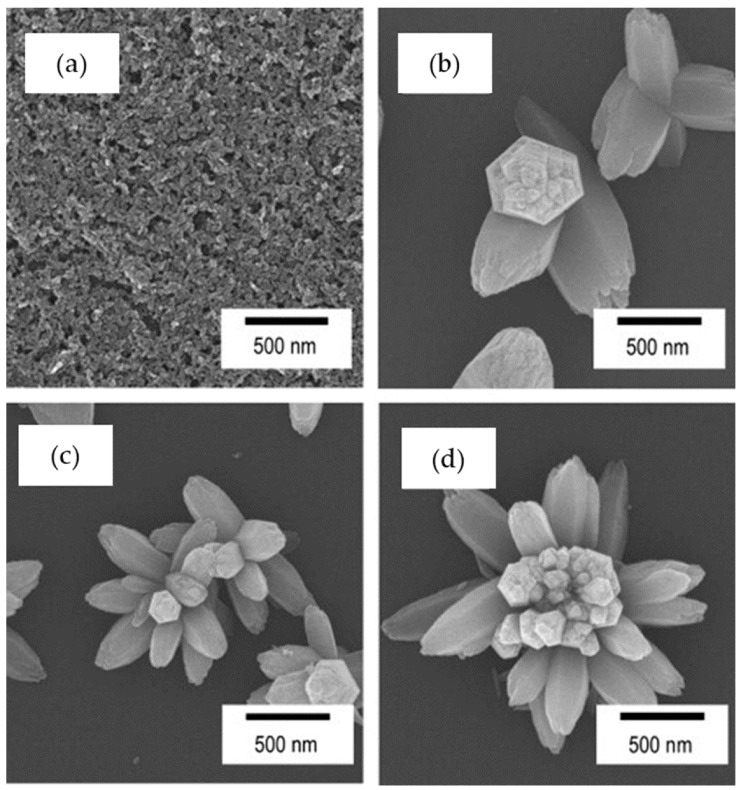
FESEM images showing the surface morphologies of the ZnO crystallites formed at the Si substrate negative electrodes after hot-water treated at 50 °C for 3 h with an applied external field of (**a**) 0 V, (**b**) 5, (**c**) 10, and (**d**) 20 V/cm. Reprinted with permission from [33], copyright (2013) Elsevier.

Zinc oxide (ZnO) is one of the extensively investigated transparent conductive oxides owing to its high exciton binding energy (60 mEV) and wide band gap of 3.37 eV [36,49,50]. These properties allow ZnO to be used in various applications, such as optical waveguides, piezoelectrics, conductive gas sensors, transparent conductive electrodes, photocatalysts, and DSSCs [51]. A comprehensive review on ZnO thin film formation by the sol–gel method was reported by Znaidi [52]. The effects of parameters such as sol concentration [53], doping [54,55,56], sol aging [57], sol water content [58], and deposition temperature [59] on the optical and electrical properties of sol–gel derived ZnO thin films have been reported. Then, Matsuda et al., demonstrated the control of ZnO crystallite morphology during a HWT at a low-temperature of 50 °C of ZnO gel films by applying an electric field [33]. The effects of time, voltage, and substrates (FTO glass and Si wafer) were investigated. On FTO substrates, the ZnO crystallites transformed from granular to columnar hexagonal structures when an electric field was applied. Interestingly, as for the ZnO sol–gel derived coating on a Si wafer, flowerlike hexagonal ZnO nanostructures were obtained when an electric field was applied. The application of the electric field influenced the orientation of the ZnO crystals, leading to the formation of this unique morphology, and the branching level increased when higher voltage was applied, as shown in Figure 9.

**Figure 10:**
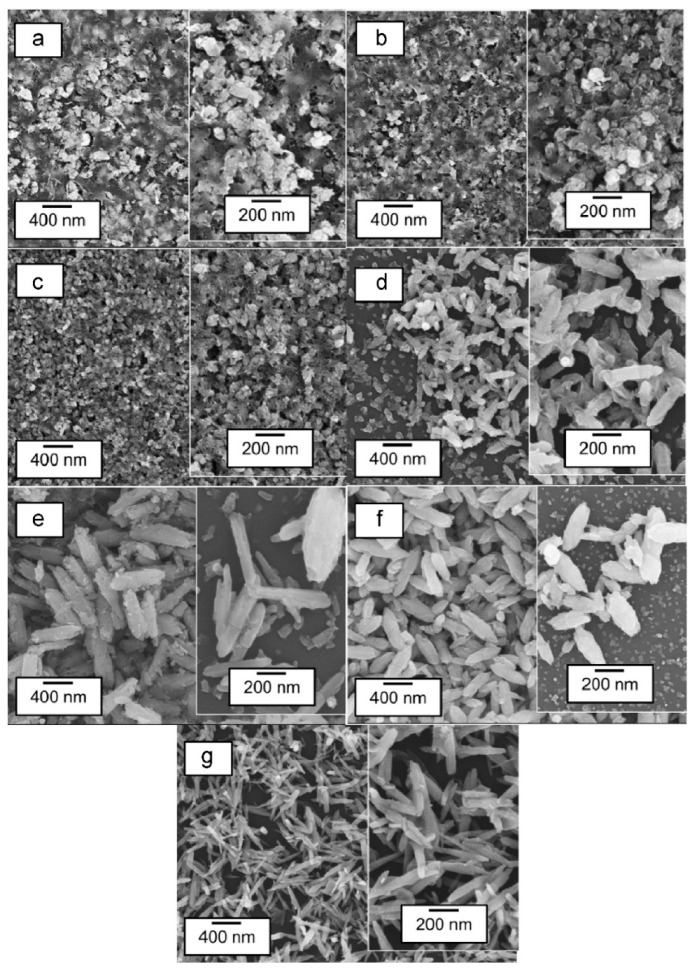

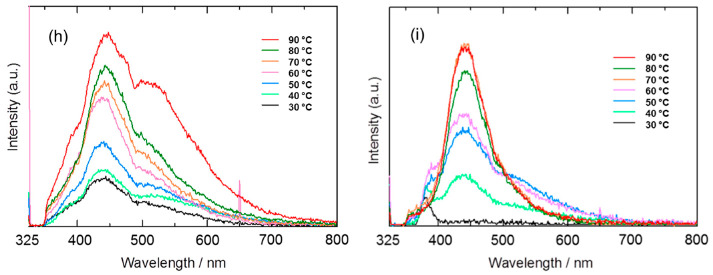
(**a**) SEM images of ZnO nanostructures formed by HWT of the sol–gel derived coatings for 1 h at (**a**) 30 °C, (**b**) 40 °C, (**c**) 50 °C, (**d**) 60 °C, (**e**) 70 °C, (**f**) 80 °C and (**g**) 90 °C. The corresponding photoluminescence spectra of the hot-water-treated sol–gel derived coatings for 1 h from 30 °C to 90 °C are represented in (**h**) as hot-water treated and (**i**) HWT and followed by heat treatment at 400 °C for 1 h. Reprinted with permission from [49], copyright (2015) Elsevier.

In a different study, Tan et al., investigated the effects of HWT temperature (30 °C to 90 °C for 1 h) on ZnO nanostructures, as well as their photoluminescence properties. With increasing HWT temperature from 30 °C to 90 °C, the morphology of the ZnO crystals transformed from spherical, rod-like, and finally needle-like nanostructures, as shown in Figure 10a–g. During the HWT, repetitive dissolution and re-deposition occurred, resulting in lattice and surface defect generation within the ZnO nanostructures. This led to the observation of prominent blue and suppressed green photoluminescence properties. Although the relative photoluminescence intensity in the blue region reduced after heat treatment at 400 °C for 1 h from a reduction of oxygen vacancies, it is noteworthy that the blue photoluminescence could still be observed, as indicated in Figure 10h,i [49].

#### 3.3.5. Formation of Layered-Double Hydroxide Films by HWT of Al_2_O_3_-Based Sol–Gel Derived Coatings

**Figure 11:**
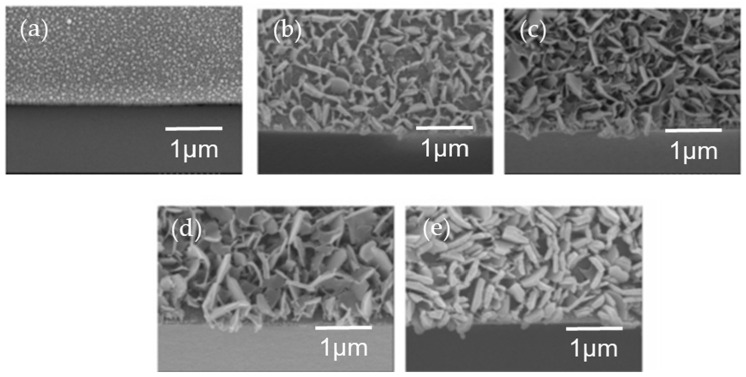
Tilted cross-sectional FESEM images (20°) of Al_2_O_3_–ZnO film (Zn/Al = 1) coated on soda lime glass substrates after (**a**) heat treatment at 400 °C (without HWT), followed by HWT at 65 °C for (**b**) 1 min, (**c**) 15 min, (**d**) 3 h, and (**e**) 4 h. Reprinted with permission from [61], copyright (2006) ACS.

The use of HWT in the development of layered double hydroxide (LDH) has also been reported. Yamaguchi et al., first discovered the formation of platelet Zn-Al-layered double hydroxide (LDH) by the HWT of heat-treated (400 °C, 30 min) Al_2_O_3_–ZnO sol–gel thin films that were prepared using aluminum tri-sec-butoxide (Al(O-sec-Bu)_3_) and zinc acetate dihydrate (Zn(OAc)_2_·2H_2_O) [60]. The Zn-Al–LDH precipitated films were synthesized directly on glass substrates after HWT, demonstrating an easy way to achieve the immobilization of Zn-Al LDH. However, the large LDH precipitates that were formed caused visible light scattering and the Zn-Al LDH films turned opaque after a short immersion time of merely 1 min. In a subsequent study, they tried to control the precipitation process to obtain transparent LDH films that were favorable for the optical property characterization of anion-intercalated LDH [61]. The surface morphologies varied with different Zn/Al ratios, and the largest amount of hexagonal structured precipitates was obtained when an equimolar of Zn/Al was used. By lowering the HWT temperature from 100 °C to 65 °C, smaller crystal precipitates were obtained, as shown in Figure 11. Even after 3 h of HWT, the obtained transmittance was higher than 50% indicating a possible control for obtaining an almost transparent LDH-precipitated films.

Recently, Tadanaga et al., further demonstrated the possible formation of intercalated Zn-Al-layered double-hydroxide films with Eosin Y by the HWT of the sol–gel derived films in distilled water containing 5 mM Eosin Y at 100 °C for 30 min [62]. The intercalation with Eosin Y improved the thermal stability of the films compared to those with Eosin Y adsorbed on the LDH surface. This was because the Zn-Al LDH provided a protective layer for Eosin Y against the oxidation by the atmospheric oxygen compared with the barely exposed Eosin Y that were adsorbed on the surface of Zn-Al LDH. They also demonstrated that the Eosin Y-intercalated Zn-Al LDH could be used as a dye-sensitized solar cell under visible light irradiation.

## 4. Future Outlook for the Sol–Gel Method in Emerging Technologies

### 4.1. Energy Storage and Conversion Applications

#### 4.1.1. Fuel Cell Technology

With the ever-increasing demand for energy and to achieve sustainability in energy sources, green technologies such as efficient batteries, supercapacitors, and fuel cells have gained tremendous interest. However, the sol–gel method is also regarded as one of the possibilities for the large-scale energy production, storage, and conversion applications.

In fuel-cell applications, proton conductivity of the electrolyte is an important factor toward achieving a stable proton supply. Daiko et al., reported on the usage of a layer-by-layer assembly to deposit poly(diallyldimethylammonium chloride (PDDA) and Nafion^TM^ onto sol–gel derived PhSiO_3/2_ microparticles [63]. A stable monolithic layer of PDDA/Nafion^TM^ -multilayer-coated phenyl-silsesquioxane (PhSiO_3/2_)was obtained after pressing the composite particles, which demonstrated proton conductivities with approximately four orders of magnitude improvement at approximately 10^−5^ S/cm at 80 °C and 90% relative humidity compared to those without [63].

Nbelayim et al., further demonstrated the applicability of the sol–gel route in the formation of Pt/TiO_2_ core–shell NPs as an electrocatalyst for a polymer electrolyte membrane fuel cell application [64]. Because Pt cores suffer from leaching, corrosion, and poisoning effects, TiO_2_ with good chemical and electrical stability was used as the protective and supportive shell layer. By using a combined micro-emulsion, sol–gel and HWT, Pt/TiO_2_ core–shell NPs with a uniform size and shape were obtained. The sizes of the Pt core and the fabricated TiO_2_ shells were approximately 6.5 nm and 0.5 nm, respectively. Most importantly, the Pt/TiO_2_ core–shell NPs exhibited the highest stability performance in fuel cell test, which is comparable to a commercial catalyst with power generations of 239, 239, and 257 mW/cm^2^ at 150 °C using Pt, Pt@TiO_2_, and a commercial catalyst, respectively.

#### 4.1.2. Photo-Electrochemical Conversion Technology

As for solar cell applications, the sol–gel route is also used to form the active layer or the required seed/buffer layer for photo-electrochemical solar-to-electric conversion, especially in DSSC applications [65,66,67,68,69,70,71,72]. Zinc oxide is one of the transparent conductive oxides. It has excellent transparency and conductivity and can be applied to photo-electrode for DSSCs [67,69,73]. The easy controllability of the ZnO morphologies is an added advantage, and the sol–gel method is one of the techniques used in the design of functional ZnO nanostructures [52,74,75,76,77]. It has been demonstrated that by using ZnO sol–gel derived coatings, nanoarchitecture modification can be achieved by low-temperature processes, such as HWT [33,36,49,78,79,80] and hydrothermal treatment [68,81,82].

Since the report on DSSCs using a TiO_2_ nanoporous structure by Regan and Gratzel in 1991, the development of TiO_2_ as a photo-electrode for DSSCs has been increasing [83]. In a recent study, the formation of pristine and Nb-doped TiO_2_ NPs and nanotubes was reported by Tsvetkov et al., using the sol–gel method [84]. Interestingly, they demonstrated that the obtained TiO_2_ nanotubes exhibited a 65% higher photoconversion efficiency when the light intensity was reduced from 1000 to 10 W/m^2^, indicating good potential in low-light conditions, such as indoor photovoltaic applications. This shows that the potential of sol–gel technology for photo-electrochemical materials design and fabrication is vast and can play a crucial role toward affordable DSSCs.

#### 4.1.3. Batteries

With the exponential demand for portable electronic devices, the requirement for lightweight and high-capacity batteries has also increased significantly. The sol–gel method is envisaged to play an important role in the future development of batteries types such as Li-ion and metal–air because it can be applied to the active material fabrication used in the anodes [85], cathodes [86], and separators [87], as well as solid-state electrolytes [88,89,90,91].

For the anode fabrication of a Li-ion battery, Mosa et al., reported on the in-situ synthesis of Li-Ti double alkoxide in their synthesis of mesoporous nanocrystalline carbon-doped Li_4_Ti_5_O_12_ thin-film with a pure spinel structure. Conductive carbon generated from the employed poly-isobutylene not only assisted in maintaining the mesoporous structure but also improved the electrical conductivity of the Li_4_Ti_5_O_12_ framework [92]. This shows that the tunability of porosity, crystallinity, and interconnectivity, which are important factors in battery performance enhancement, can be achieved and controlled using the sol–gel method.

**Figure 12:**
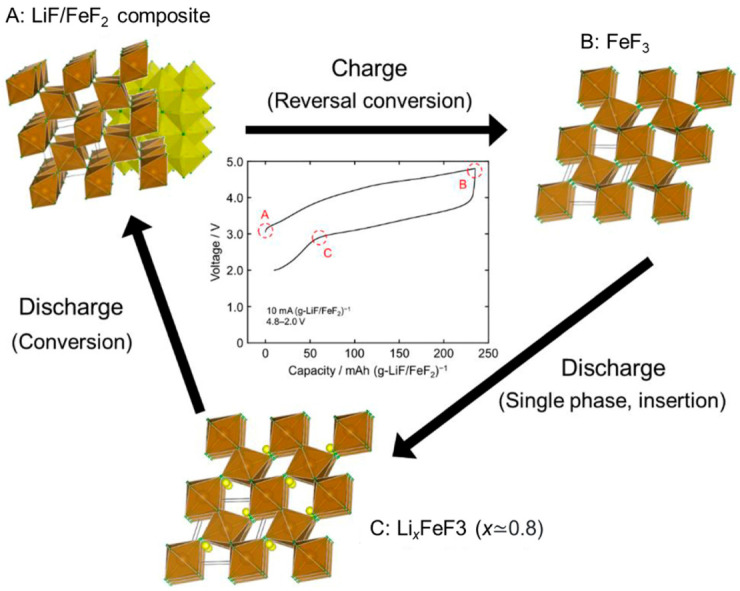
Schematic of the reaction mechanism of the LiF/FeF_2_ composite prepared by the fluorolytic sol–gel method (brown octahedron: FeF_6_, yellow octahedron: LiF_6_, green circle: F, yellow circle: Li). During the charge-discharge process (A–C), the conversion reaction to LiF and FeF_2_ follows Li^+^-insertion to FeF_3_. Reprinted with permission from [86], copyright (2019) Elsevier.

As for the cathode materials of a Li-ion battery, a LiF/FeF_2_ nanocomposite prepared using ball-milling was reported to exhibit a large reversible capacity of 190 mAh g^−1^, with a reversible charge–discharge reaction proposed to occur through the formation of Li0.5FeF_3_ [93]. However, this technique is inappropriate for mass production and has a high tendency to cause contamination. Recently, Tawa et al., reported a new charge–discharge mechanism using a nanocomposite that consisted of LiF and FeF_2,_ which was obtained using the fluorolytic sol–gel method with an excellent potential for production scale-up, as shown in Figure 12 [86]. The obtained LiF/FeF_2_ exhibited an initial reversible capacity of 225 mAh (g-LiF/FeF_2_)^−1^at a current rate of 10 mA (g-LiF/FeF_2_)^−1^, which is comparable to the theoretical capacity of 227 mAh (g-LiF/FeF_2_)^−1^. These recent studies demonstrated the applicability and potential of the sol–gel method to produce advanced materials with novel functionalities for Li-ion batteries.

### 4.2. Superhydrophobic/Superhydrophilic Layer Formation

Through surface design, coatings and films that possess hydrophobicity or hydrophilicity can be used for self-cleaning and in liquid mixture separation technology [94]. Tadanaga et al., have reported on the formation of convex-shaped oxide micropatterns using hydrophobic–hydrophilic patterned surfaces for the formation of ZrO_2_, TiO_2_, and Al_2_O_3_ patterned films by the hydrolysis of ZrCl_4_, TiCl_4_, and AlCl_3_ dissolved in ethanol, respectively [95]. This shows that the sol–gel method can play an important role in the formation of hydrophobic–hydrophilic–patterned surfaces as well as patterned oxide layers through the hydrolysis of alkoxides, which offers an advantage over physical processing methods in term of cost and scalability for optical component manufacturing.

**Figure 13:**
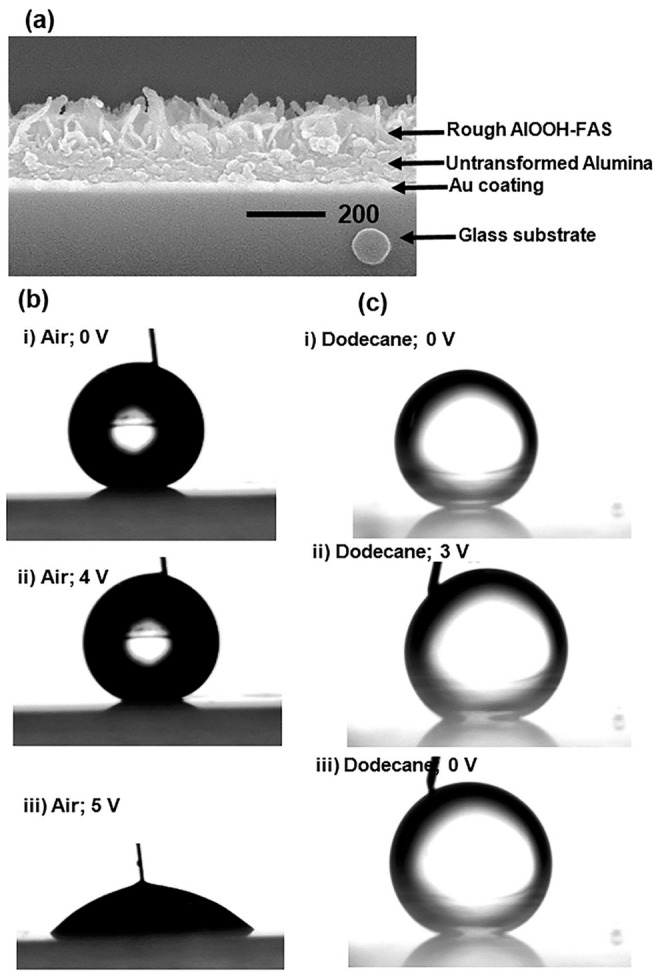
(**a**) FESEM cross-sectional image of sample for EW & REW; droplet images of (**b**) EW in air and (**c**) REW in dodecane medium. (**b**(**i**)) sessile droplet with an inserted gold (Au) conductor with 0 V supply, CA = 155.4 ± 0.6°; (**b**(**ii**)) 4 V supply, CA = 153.3 ± 0.4°; (**b**(**iii**)) 5 V supply, CA = 48.5 ± 1.2°. (**c**(**i**)) droplet with 0 V, CA = 161.3 ± 0.4°; (**c**(**ii**)) droplet with 3 V supply, CA = 152.4 ± 0.6°; (**c**(**iii**)) reversed electro-wetted droplet when voltage was turned off, CA = 157.3 ± 0.8°. Reprinted with permission from [96], copyright (2017) Elsevier.

Following the discovery of Tadanaga et al., new developments in the superhydrophobic behavior of robust pseudoboehmite alumina composites, as well as their electrowetting properties using a low actuation voltage in air and dodecane, were reported by Nbelayim et al. [96]. The electrowetting properties of the nanocoating obtained using sol–gel and HWT are demonstrated in Figure 13 showing reverse electrowetting behavior. This shows that the sol–gel method can be used in films fabrication for nanoscale electrowetting devices.

### 4.3. Formation of Porous Ceramics Films

The sol–gel method is also commonly used in the fabrication of three-dimensional porous ceramic networks [97]. Microporous and mesoporous ceramics are widely used in adsorption technology and drug delivery, resulting in rapid development in this category of materials [98,99]. The formation of porous ceramics such as ZrO_2_ [5,6], SiC [100], Al_2_O_3_ [101], and SiO_2_ wrinkle [102], as well as core-shell composites, has been reported [98,102,103,104].

Zirconium oxide is a material of interest because of its good chemical stability in sensor applications and as an adsorbent. Using Pluronic 123 as a template in the sol–gel route, the formation of either amorphous, tetragonal, or monoclinic ZrO_2_ can be achieved by adjusting the annealing temperature [5]. It has also been further reported that a hierarchical pore structure formation can be obtained by adjusting the film deposition using the Pluronic 123 templating method. The controllability of ZrO_2_ film formation, in terms of thickness and porosity (meso–macroporous level), by adjusting the spin-coating speed and coating layer, is well described by Soo et al. The co-existing microstructure, which consisted of worm-like mesopores and macropores with good optical transmittance in the visible region, had good potential for applications such as sensing and catalysis [6].

The formation of hollow structured ceramic spheres has also attracted widespread interest due to their potential for biomedical drug delivery and cosmetic application [105]. Katagiri et al., reported on a simple, one-step fabrication of hollow TiO_2_ and strontium titanate (SrTiO_3_) nanospheres [98]. The one-step process involved the simultaneous shell crystallization and core dissolution during hydrothermal treatment forming hollow nanospheres with an average diameter of 200 nm. In their core-shell particle formation, monodispersed silica gel particles were first formed using sol–gel method followed by layer-by-layer (LbL) coating of titanium (IV) bis(ammonium lactato)dihydroxide (TALH) precursor (also act as weak polyelectrolyte) with poly(sodium p-styrene sulfonate) (PSS) and poly(diallyl dimethylammonium chloride) (PDDA) polyelectrolytes. LbL assembly using electrostatic interaction of polyelectrolytes enables homogenous deposition of the TALH precursor on the surface of silica gel nanospheres [106,107]. Finally, hydrothermal treatment using water or strontium hydroxide aqueous solution would determine the formation either hollow TiO_2_ or hollow SrTiO_3_ nanospheres, respectively.

### 4.4. Extraction Technology for Waste Removal

With the rapid industrial development needed to satisfy the consumer demand, a significant amount of waste has been created, which has also contaminated our sources of water. One major contaminant found in plastic materials is bisphenol A (BPA), which is widely used in food containers, bottles, and medical equipment. Owing to the structural similarity of BPA with natural hormones, it can disrupt the endocrine formation and is regarded as a health risk. Kalogiouri et al., recently reported on the synthesis of molecularly imprinted polymers using sol–gel matrix imprinting for the extraction of BPA solids from water [108]. The matrix exhibited high selectivity and high absorption capability with a reusability of up to 10 times while maintaining extraction performance.

**Figure 14:**
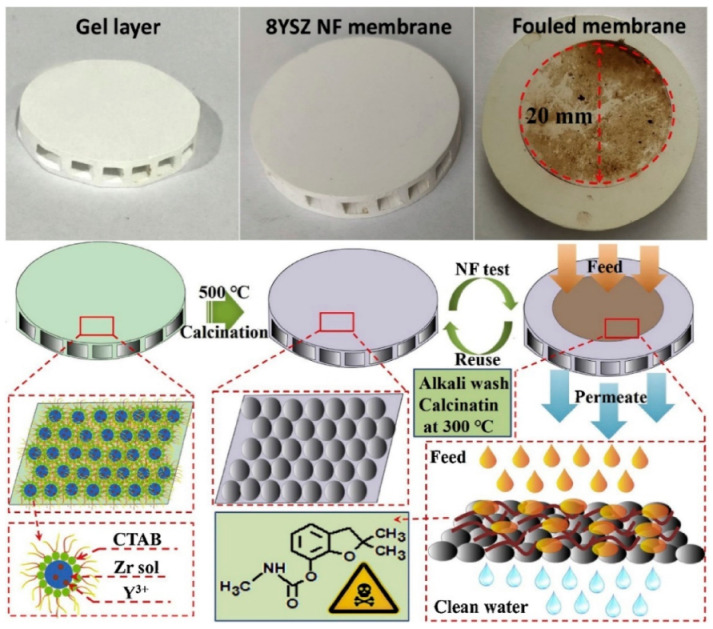
Procedure for 8-mol% yttria-stabilized ZrO_2_ nanofiltration membranes formation by reverse micelles-mediated sol–gel process and carbofuran removal. Reprinted with permission from [112], copyright (2020) Elsevier.

In wastewater treatment, the removal of heavy metals from a water source is crucial, owing to the serious health hazards and long-lasting complications that these contaminants pose to humans [109]. The sol–gel method has been used for novel material fabrication for heavy metal removal and adsorption, such as sol–gel derived, electro-spined flexible Fe_3_O_4_ fibers [110], mesoporous TiO_2_ with sodium-modified surfaces [111], sol–gel ultrafine alumina fibers [15] and yttria-stabilized ZrO_2_ membranes [112]. A recent study by Qin et al., demonstrated the fabrication of yttria-stabilized ZrO_2_ nanofiltration membranes. They used size-controlled spherical ZrO_2_ NPs through via a reverse micelles-mediated sol–gel process for pesticide removal from water, as shown in Figure 14 [112]. The doping of yttria suppressed tetragonal-to-monoclinic phase transition, improving membrane integrity. Importantly, this reduced the tetragonal grain size, which improved the specific surface area and led to a better nanofiltration process. In their carbofuran removal test, a high removal rate of 89% was achieved. The above-mentioned studies consolidate the importance of sol–gel derived materials for waste removal technologies, which are crucial for the achievement of sustainable development goals involving water resources.

### 4.5. Templates for Plasmonic Effect Generation

The limited light adsorption of photocatalytic materials such as ZnO and TiO_2_ in the UV region amounts to merely 3–5% of solar radiation. Therefore, researchers have used the deposition of metal NPs onto oxide films to enhance adsorption in the visible light region through surface plasmon resonance effect [66,67,69,70,71,72]. Importantly, the photocatalytic property is largely dependent on the surface area of the material, and a mesoporous template is commonly used for the decoration of metal NPs to maximize the plasmon resonance effect. The formation and engineering of a mesoporous template can be achieved using the sol–gel method to obtain a desired template prior to the deposition of plasmonic nanostructures. 

Using a SiO_2_–TiO_2_ sol–gel template, Okuno et al., reported on the deposition of Au NPs and Au nanorods (NRs) by photodeposition into the tubular mesochannels template, forming Au/SiO_2_–TiO_2_ composites [113]. They found that the photocatalytic properties of Au-NRs/SiO_2_–TiO_2_ were the highest in methylene blue degradation compared with Au-NPs/SiO_2_–TiO_2_ and standard TiO_2_ P25 films. This was due to the following two factors; (i) the high surface area needed for the formation of the Schottky barrier between the Au and TiO_2_ using the sol–gel derived template and, (ii) the tubular mesochannels worked as a template for the formation/deposition of Au NRs, which absorbed visible and near-infrared light energy used for photocatalysis [113]. Their findings demonstrated that the sol–gel route could be useful for porous template formation in plasmonic composite formation.

Besides, core-shell NPs are also reported to be able to capitalize on the local surface plasmon effect to achieve an enhanced photo-electrochemical conversion efficiency. Nbelayim et al. used the sol–gel route in the formation of Ag@TiO_2_ core-shell NPs for DSSC applications. They obtained improved efficiency owing to the plasmon effect of Ag, which enabled improved light adsorption and modification of the Fermi level of the TiO_2_ photo-anode for efficient charge injection [71]. These developments illustrate the possible usage of sol–gel derived templates for plasmonic material decoration to achieve improved photocatalytic properties. 

### 4.6. Nanocomposites Formation via Sol–Gel Templating

**Figure 15:**
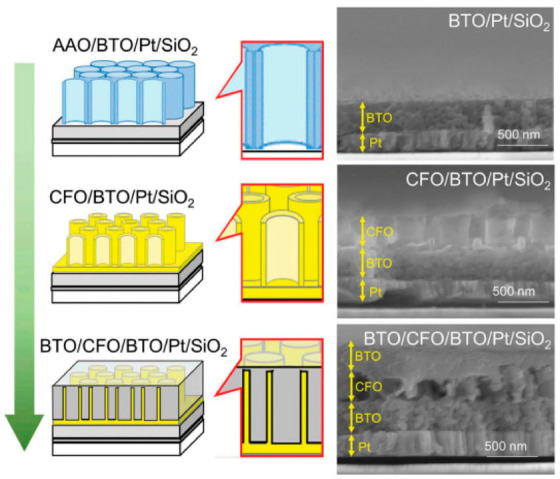
Schematic of the fabrication process for the multiferroic nanocomposite, BTO/CFO/BTO, on a Pt-coated SiO_2_ substrate. The corresponding SEM images are shown on the right. From top to bottom: (anodic alumina oxide (AAO)-placed) BTO compact layer on a Pt-coated SiO_2_ substrate; CFO nanotube arrays on BTO/Pt/SiO_2_; BTO coated CFO nanotube arrays on BTO/Pt/SiO_2_. Reprinted with permission from [117], copyright (2018) Taylor and Francis.

Multiferroic nanocomposites are composites that exhibit both ferromagnetism and ferro-electricity with potential applications in data storage devices and microwave transducers [114]. Owing to the high cost of aligned composite formation using physical methods, researchers have shifted toward a liquid phase method with the aim of producing affordable multiferroic devices. The use of the sol–gel method has recently gained pace for film-type, templated multiferroic films as well as heterostructure composite formation [115,116]. In the formation of BaTiO_3_ (BTO)-CoFe_2_O_4_ (CFO) multiferroic composite, Kawamura et al., used the sol–gel route in the fabrication, as follows: BaTiO_3_ (BTO) (ferroelectric) was first sol–gel spin-coated on a Pt-deposited SiO_2_ wafer [117]. Then, using anodic alumina oxide (AAO) as a template, the sol–gel spin-coating of a CoFe_2_O_4_ (CFO) (ferrimagnetic) was carried out. Next, after etching of the alumina with NaOH, CFO nanotubes were obtained. Finally, another layer of BTO was spin-coated on top to fabricate a composite structure that consisted of ferrimagnetic nanopillars embedded in a ferroelectric matrix, as shown in Figure 15. The multiferroicity property of the BTO-CFO composite was confirmed exhibiting dielectric hysteresis loops which was controllable by an external magnetic field. 

**Figure 16:**
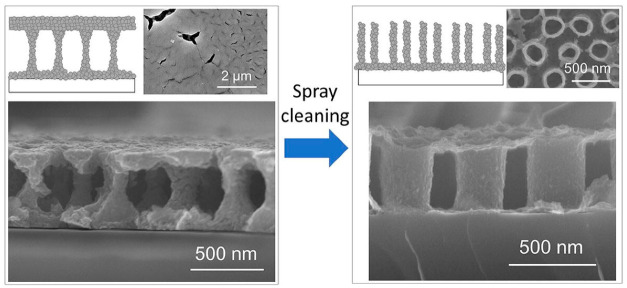
Novel nano-periodic structured BaTiO_3_ films prepared by a sol–gel template synthesis using a through-hole-type AAO template film. The nano-periodic structure was drastically modified from a liquid-bridge-like structure to nanotube arrays by incorporating a spray-cleaning step with 2-methoxyethanol during spin-coating of the template. Reprinted with permission from [118], copyright (2018) Elsevier.

In a different study, they further demonstrated the feasibility forming nano-periodic porous structures using sol–gel template synthesis. Nano-periodic structure refers to a regular and repetitive arrangement of a nanometer-sized unit structure, in this case, liquid-bridge and nanotube structures. A spray-cleaning step with 2-methoxyethanol was incorporated during the spin-coating process of the AAO template with a BTO sol. As a result, drastic modification of the film’s microstructure from a liquid-bridge-like structure to nanotube arrays occurred, as shown in Figure 16 [118]. Owing to the dependency of piezo-electricity on the porosity and nanopore arrangement, the unique nano-periodic BTO films obtained this templating method allowed better piezo-electric behavior control. These films can be used in nanocomposites for micro-electromechanical systems [119], multiferroics [120,121], and bone tissue engineering [122].

### 4.7. Formation of Unique and Hierarchical Composite Structures

Using hybrid organic-inorganic materials fabricated via the sol–gel route, Takahashi described and reviewed on the formation of responsive and adaptive micro wrinkles through buckling stimulation [123]. The wrinkled films generated can be used as template for nanoscale materials alignment and ordering, enabling the control of optical and electronic properties. Moreover, the nano- and microscale periodicity can also be used for wettability control, micro patterning, size-selective adsorption/desorption of functional targets (cells or microorganisms), micro-rolls formation, stretchable electronic substrates, and soft actuators [124,125]. 

**Figure 17:**
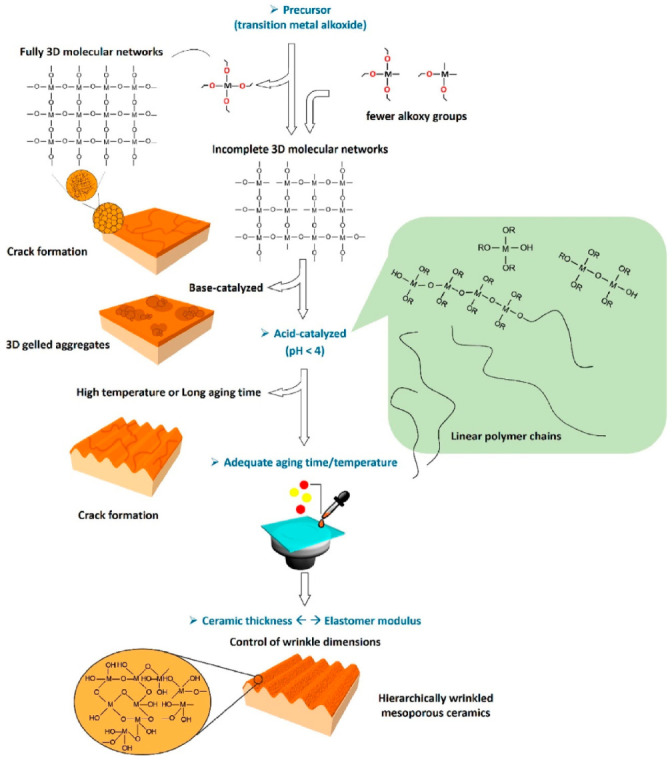
Illustration of the creation of hierarchically wrinkled mesoporous ceramic with controllable geometric dimensions through the combination of sol–gel reactions and surface wrinkling. Reprinted with permission from [102], copyright (2021) Elsevier.

In an interesting recent development, it was demonstrated that hierarchical ceramic structures, which require sophisticated and expensive equipment, could be fabricated using the sol–gel route. Xie et al. constructed hierarchical wrinkled mesoporous ceramic surfaces on soft elastomer using a dynamic interfacial release process [102]. They did this using ceramic thin films produced from interfacial sol–gel reactions and elastomer cross-linking contraction for surface wrinkling. The wrinkled ceramic, which consisted of SiO_2_/PDMS, was used for cell alignment because the mesoporous wrinkled nanostructures promoted cell adhesion and enhanced cell growth in an aligned manner, as shown in Figure 17. The figure demonstrates that by controlling the sol–gel reactions, the formation of hierarchically wrinkled mesoporous ceramic with the desired geometric dimensions can be obtained. This indicates that the cost-effective sol–gel derived method can be used for mesoporous wrinkled structure fabrication in biomedical applications.

## 5. Conclusions

The sol–gel method is a versatile technique for the fabrication of organic and inorganic, as well as hybrid, nanomaterials. From the perspective of cost, simplicity, and scalability, the sol–gel route offers many advantages, flexibility, and tunability to create the desired functional materials. It has been demonstrated that simple sol–gel modification techniques such as electrophoretic patterning, photoirradiation, and HWT can be used to alter and control the structure as well as crystallinity of the coatings. Furthermore, the feasibility of low-temperature crystallization, by lowering the calcination temperature or by HWT, not only reduced the energy consumption but also enabled its application on polymeric substrates with low thermal stability with potential for flexible device applications. The vast potential of sol–gel technology for advanced applications such as energy storage and conversion as well as fabrication of novel composites are emphasized, demonstrating a good outlook of this technique for materials development. Despite the rapid development and numerous reported works, the sol–gel method is still regarded as one of the important methods for novel advanced material fabrication for new emerging technologies. Although it is impossible to cover all the works related to the sol–gel method, this review provides a brief revisit of the progress and recent developments that underline the significant potential of the method in the creation of advanced functional materials toward sustainable development goals.

## Data Availability

Restrictions apply to the availability of these data. Some data are not publicly available since some articles are not open access.

## References

[1] Matsuda A., Kawamura G. (2016). Sol–Gel Nano-/Micropatterning Process. Handbook of Sol-Gel Science and Technology.

[2] Matsuda A., Tatsumisago M. (2016). Electrophoretic Sol–Gel Deposition. Handbook of Sol-Gel Science and Technology.

[3] Ulrich D.R. (1988). Prospects of sol-gel processes. J. Non-Cryst. Solids.

[4] Hench L.L., West J.K. (1990). The sol-gel process. Chem. Rev..

[5] Soo M.T., Kawamura G., Muto H., Matsuda A., Lockman Z., Cheong K.Y. (2013). Fabrication of well-crystallized mesoporous ZrO_2_ thin films via Pluronic P123 templated sol–gel route. Ceram. Int..

[6] Soo M.T., Kawamura G., Muto H., Matsuda A., Lockman Z., Cheong K.Y. (2013). Design of hierarchically meso–macroporous tetragonal ZrO_2_ thin films with tunable thickness by spin-coating via sol–gel template route. Microporous Mesoporous Mater..

[7] Soo M.T., Prastomo N., Matsuda A., Kawamura G., Muto H., Noor A.F.M., Lockman Z., Cheong K.Y. (2012). Elaboration and characterization of sol–gel derived ZrO_2_ thin films treated with hot water. Appl. Surf. Sci..

[8] Aurobind S.V., Amirthalingam K.P., Gomathi H. (2006). Sol-gel based surface modification of electrodes for electro analysis. Adv Colloid Interface Sci..

[9] Nisticò R., Scalarone D., Magnacca G. (2017). Sol-gel chemistry, templating and spin-coating deposition: A combined approach to control in a simple way the porosity of inorganic thin films/coatings. Microporous Mesoporous Mater..

[10] Sakka S., Klein L., Aparicio M., Jitianu A. (2016). The Outline of Applications of the Sol–Gel Method. Handbook of Sol-Gel Science and Technology.

[11] Kessler V.G., Klein L., Aparicio M., Jitianu A. (2018). The Synthesis and Solution Stability of Alkoxide Precursors. Handbook of Sol-Gel Science and Technology: Processing, Characterization and Applications.

[12] Amri A., Jiang Z.T., Pryor T., Yin C.-Y., Djordjevic S. (2014). Developments in the synthesis of flat plate solar selective absorber materials via sol–gel methods: A review. Renew. Sustain. Energy Rev..

[13] Jittiarporn P., Badilescu S., Al Sawafta M.N., Sikong L., Truong V.-V. (2017). Electrochromic properties of sol–gel prepared hybrid transition metal oxides—A short review. J. Sci. Adv. Mater. Devices.

[14] Ismail W.N.W. (2016). Sol–gel technology for innovative fabric finishing—A Review. J. Sol-Gel Sci. Technol..

[15] Li L., Liu X., Wang G., Liu Y., Kang W., Deng N., Zhuang X., Zhou X. (2020). Research progress of ultrafine alumina fiber prepared by sol-gel method: A review. Chem. Eng. J..

[16] Brinker C.J., Frye G.C., Hurd A.J., Ashley C.S. (1991). Fundamentals of sol-gel dip coating. Thin Solid Films.

[17] Salvaggio M.G., Passalacqua R., Abate S., Perathoner S., Centi G., Lanza M., Stassi A. (2016). Functional nano-textured titania-coatings with self-cleaning and antireflective properties for photovoltaic surfaces. Sol. Energy.

[18] Boccaccini A.R. (2010). Electrophoretic deposition: Fundamentals and applications. J. Eur. Ceram. Soc..

[19] Chávez-Valdez A., Boccaccini A.R. (2012). Innovations in electrophoretic deposition: Alternating current and pulsed direct current methods. Electrochim. Acta.

[20] Corni I., Ryan M.P., Boccaccini A.R. (2008). Electrophoretic deposition: From traditional ceramics to nanotechnology. J. Eur. Ceram. Soc..

[21] Takahashi K., Tadanaga K., Hayashi A., Tatsumisago M., Matsuda A. (2006). Micropatterning of Transparent Poly(Benzylsilsesquioxane) Thick Films Prepared by the Electrophoretic Sol-Gel Deposition Process Using a Hydrophobic?Hydrophilic-Patterned Surface. J. Am. Ceram. Soc..

[22] Takahashi K., Tadanaga K., Matsuda A., Hayashi A., Tatsumisago M. (2006). Formation of convex shaped poly(phenylsilsesquioxane) micropatterns on indium tin oxide substrates with hydrophobic-hydrophilic patterns using the electrophoretic sol-gel deposition method. J. Mater. Res..

[23] Takahashi K., Tadanaga K., Tatsumisago M., Matsuda A. (2006). Characterization and Electrophoretic Deposition of Poly(Phenylsilsesquioxane)-Titania Hybrid Particles Prepared by the Sol-Gel Method. J. Am. Ceram. Soc..

[24] Takahashi K., Tadanaga K., Matsuda A., Hayashi A., Tatsumisago M. (2007). Fabrication of convex-shaped polybenzylsilsesquioxane micropatterns by the electrophoretic sol–gel deposition process using indium tin oxide substrates with a hydrophobic-hydrophilic-patterned surface. J. Sol-Gel Sci. Technol..

[25] Tohge N., Shinmou K., Minami T. (1994). Effects of UV-irradiation on the formation of oxide thin films from chemically modified metal-alkoxides. J. Sol-Gel Sci. Technol..

[26] Tadanaga K., Owan T., Morinaga J., Urbanek S., Minami T. (2000). Fine Patterning of Transparent, Conductive SnO2 Thin Films by UV-Irradiation. J. Sol-Gel Sci. Technol..

[27] Kawamura G., Sato S., Muto H., Sakai M., Lim P.B., Watanabe K., Inoue M., Matsuda A. (2010). AgBr nanocrystal-dispersed silsesquioxane–titania hybrid films for holographic materials. Mater. Lett..

[28] Kawamura G., Tsurumi Y., Muto H., Sakai M., Inoue M., Matsuda A. (2011). Reversible conversion between AgCl and Ag in AgCl-doped RSiO3/2–TiO_2_ films prepared by a sol–gel technique. Mater. Chem. Phys..

[29] Kotani Y., Matsuda A., Tatsumisago M., Minami T., Umezawa T., Kogure T. (2000). Formation of Anatase Nanocrystals in Sol-Gel Derived TiO_2_-SiO_2_ Thin Films with Hot Water Treatment. J. Sol-Gel Sci. Technol..

[30] Matsuda A., Kotani Y., Kogure T., Tatsumisago M., Minami T. (2000). Transparent Anatase Nanocomposite Films by the Sol–Gel Process at Low Temperatures. J. Am. Ceram. Soc..

[31] Matsuda A., Matoda T., Kogure T., Tadanaga K., Minami T., Tatsumisago M. (2005). Formation and Characterization of Titania Nanosheet-Precipitated Coatings via Sol−Gel Process with Hot Water Treatment under Vibration. Chem. Mater..

[32] Prastomo N., Daiko Y., Kogure T., Muto H., Sakai M., Matsuda A. (2009). Formation mechanism of titania nanosheet cryatallites on silica–titania gel films by vibration hot-water treatment. Mater. Sci. Eng. B.

[33] Matsuda A., Tan W.K., Furukawa S., Muto H. (2013). Morphology-control of crystallites precipitated from ZnO gel films by applying electric field during hot-water treatment. Mater. Sci. Semicond. Process..

[34] Prastomo N., Muto H., Sakai M., Matsuda A. (2010). Formation and stabilization of tetragonal phase in sol–gel derived ZrO_2_ treated with base-hot-water. Mater. Sci. Eng. B.

[35] Prastomo N., Zakaria N.H.b., Kawamura G., Muto H., Sakai M., Matsuda A. (2011). High surface area BaZrO_3_ photocatalyst prepared by base-hot-water treatment. J. Eur. Ceram. Soc..

[36] Tan W.K., Abdul Razak K., Lockman Z., Kawamura G., Muto H., Matsuda A. (2013). Photoluminescence properties of rod-like Ce-doped ZnO nanostructured films formed by hot-water treatment of sol–gel derived coating. Opt. Mater..

[37] Kotani Y., Matoda T., Matsuda A., Kogure T., Tatsumisago M., Minami T. (2001). Anatase nanocrystal-dispersed thin films via sol–gel process with hot water treatment: Effects of poly(ethylene glycol) addition on photocatalytic activities of the films. J. Mater. Chem..

[38] Matsuda A., Matoda T., Tadanaga K., Minami T., Tatsumisago M., Kogure T. (2005). Lowering of Preparation Temperatures of Anatase Nanocrystals-Dispersed Coatings via Sol-Gel Process with Hot Water Treatment. J. Am. Ceram. Soc..

[39] Katagiri K., Harada G., Matsuda A., Kogure T., Muto H., Sakai M. (2006). Effects of Addition of Supramolecular Assembly on the Anatase Nanocrystalline Precipitation of Sol–Gel Derived SiO_2_–TiO_2_ Coating Films by Hot-Water Treatment. J. Nanosci. Nanotechnol..

[40] Matsuda A., Higashi Y., Tadanaga K., Tatsumisago M. (2006). Hot-water treatment of sol–gel derived SiO_2_–TiO_2_ microparticles and application to electrophoretic deposition for thick films. J. Mater. Sci..

[41] Prastomo N., Kimata K., Daiko Y., Muto H., Kogure T., Sakai M., Matsuda A. (2010). Effect of external fields applied during hot-water treatment on the aspect ratio of nanocrystallites formed on SiO_2_·TiO_2_ coatings derived from sol–gel techniques. J. Sol-Gel Sci. Technol..

[42] Matsuda A., Kobayashi K., Kogure T., Sakai M., Tadanaga K., Minami T., Tatsumisago M. (2008). Characterization of ramiform precipitates formed on SiO_2_–TiO_2_ gel coatings by electric field hot water treatment. J. Non-Cryst. Solids.

[43] Popovici M., de Graaf J., Verschuuren M.A., Graat P.C.J., Verheijen M.A. (2010). Zirconia thin film preparation by wet chemical methods at low temperature. Thin Solid Films.

[44] Chen D. (2001). Anti-reflection (AR) coatings made by sol–gel processes: A review. Sol. Energy Mater. Sol. Cells.

[45] Kauppinen C., Isakov K., Sopanen M. (2017). Grass-like Alumina with Low Refractive Index for Scalable, Broadband, Omnidirectional Antireflection Coatings on Glass Using Atomic Layer Deposition. ACS Appl. Mater. Interfaces.

[46] Yamaguchi N., Tadanaga K., Matsuda A., Minami T., Tatsumisago M. (2007). Antireflective properties of flowerlike alumina thin films on soda–lime silica glass substrates prepared by the sol–gel method with hot water treatment. Thin Solid Films.

[47] Yamaguchi N., Tadanaga K., Matsuda A., Minami T., Tatsumisago M. (2006). Formation of anti-reflective alumina films on polymer substrates by the sol–gel process with hot water treatment. Surf. Coat. Technol..

[48] Tadanaga K., Yamaguchi N., Uraoka Y., Matsuda A., Minami T., Tatsumisago M. (2008). Anti-reflective properties of nano-structured alumina thin films on poly(methyl methacrylate) substrates by the sol–gel process with hot water treatment. Thin Solid Films.

[49] Tan W.K., Kawamura G., Muto H., Razak K.A., Lockman Z., Matsuda A. (2015). Blue-emitting photoluminescence of rod-like and needle-like ZnO nanostructures formed by hot-water treatment of sol–gel derived coatings. J. Lumin..

[50] Valverde-Aguilar G., Manríquez Zepeda J.L. (2014). Photoluminescence and photoconductivity studies on amorphous and crystalline ZnO thin films obtained by sol–gel method. Appl. Phys. A.

[51] Djurišić A.B., Ng A.M.C., Chen X.Y. (2010). ZnO nanostructures for optoelectronics: Material properties and device applications. Prog. Quantum Electron..

[52] Znaidi L. (2010). Sol–gel-deposited ZnO thin films: A review. Mater. Sci. Eng. B.

[53] Xu L., Zheng G., Miao J., Xian F. (2012). Dependence of structural and optical properties of sol–gel derived ZnO thin films on sol concentration. Appl. Surf. Sci..

[54] Xu L., Zheng G., Zhao L., Pei S. (2015). Two different mechanisms on UV emission enhancement in Ag-doped ZnO thin films. J. Lumin..

[55] Ebrahimifard R., Golobostanfard M.R., Abdizadeh H. (2014). Sol–gel derived Al and Ga co-doped ZnO thin films: An optoelectronic study. Appl. Surf. Sci..

[56] Salam S., Islam M., Akram A. (2013). Sol–gel synthesis of intrinsic and aluminum-doped zinc oxide thin films as transparent conducting oxides for thin film solar cells. Thin Solid Films.

[57] Ibrahim N.B., Al-Shomar S.M., Ahmad S.H. (2013). Effect of aging time on the optical, structural and photoluminescence properties of nanocrystalline ZnO films prepared by a sol–gel method. Appl. Surf. Sci..

[58] Nehmann J.B., Ehrmann N., Reineke-Koch R., Bahnemann D.W. (2014). Aluminum-doped zinc oxide sol–gel thin films: Influence of the sol’s water content on the resistivity. Thin Solid Films.

[59] Caglar M., Ruzgar S. (2015). Influence of the deposition temperature on the physical properties of high electron mobility ZnO films by sol–gel process. J. Alloys Compd..

[60] Naoko Y., Tomohiko N., Kiyoharu T., Atsunori M., Tsutomu M., Masahiro T. (2006). Platelike Crystal Growth of Zn–Al Layered Double Hydroxide by Hot Water Treatment of Sol–Gel Derived Al2O3–ZnO Films on Glass Substrate. Chem. Lett..

[61] Yamaguchi N., Nakamura T., Tadanaga K., Matsuda A., Minami T., Tatsumisago M. (2006). Direct Formation of Zn−Al Layered Double Hydroxide Films with High Transparency on Glass Substrate by the Sol−Gel Process with Hot Water Treatment. Cryst. Growth Des..

[62] Tadanaga K., Oi J.-i., Higuchi M. (2016). Preparation of Zn–Al layered double hydroxide thin films intercalated with Eosin Y by hot water treatment of sol-gel derived precursor films. J. Sol-Gel Sci. Technol..

[63] Daiko Y., Sakamoto H., Katagiri K., Muto H., Sakai M., Matsuda A. (2008). Deposition of Ultrathin Nafion Layers on Sol–Gel-Derived Phenylsilsesquioxane Particles via Layer-by-Layer Assembly. J. Electrochem. Soc..

[64] Nbelayim P., Ashida Y., Maegawa K., Kawamura G., Muto H., Matsuda A. (2020). Preparation and Characterization of Stable and Active Pt@TiO2 Core–Shell Nanoparticles as Electrocatalyst for Application in PEMFCs. ACS Appl. Energy Mater..

[65] Toe M.Z., Pung S.Y., Yaacob K.A., Matsuda A., Tan W.K., Han S.S. (2020). Effect of TiO2 sol on the conversion efficiency of TiO_2_ based dye-sensitized solar cell. J. Sol-Gel Sci. Technol..

[66] Abd-Ellah M., Moghimi N., Zhang L., Thomas J.P., McGillivray D., Srivastava S., Leung K.T. (2016). Plasmonic gold nanoparticles for ZnO-nanotube photoanodes in dye-sensitized solar cell application. Nanoscale.

[67] Tan W.K., Ito T., Kawamura G., Muto H., Lockman Z., Matsuda A. (2017). Controlled facile fabrication of plasmonic enhanced Au-decorated ZnO nanowire arrays dye-sensitized solar cells. Mater. Today Commun..

[68] Tan W.K., Lockman Z., Razak K.A., Kawamura G., Muto H., Matsuda A. (2013). Enhanced dye-sensitized solar cells performance of ZnO nanorod arrays grown by low-temperature hydrothermal reaction. Int. J. Energy Res..

[69] Tan W.K., Muto H., Ito T., Kawamura G., Lockman Z., Matsuda A. (2020). Facile Fabrication of Plasmonic Enhanced Noble-Metal-Decorated ZnO Nanowire Arrays for Dye-Sensitized Solar Cells. J. Nanosci. Nanotechnol..

[70] Nbelayim P., Kawamura G., Abdel-Galeil M.M., Tan W.K., Wei X., Muto H., Matsuda A. (2018). Effects of multi-sized and -shaped Ag@TiO_2_ nanoparticles on the performance of plasmonic dye-sensitized solar cells. J. Ceram. Soc. Jpn..

[71] Nbelayim P., Kawamura G., Kian Tan W., Muto H., Matsuda A. (2017). Systematic characterization of the effect of Ag@TiO_2_ nanoparticles on the performance of plasmonic dye-sensitized solar cells. Sci. Rep..

[72] Nbelayim P., Kawamura G., Tan W.K., Muto H., Matsuda A. (2018). Ag@TiO_2_ Nanowires-Loaded Dye-Sensitized Solar Cells and Their Effect on the Various Performance Parameters of DSSCs. J. Electrochem. Soc..

[73] Sacco A., Lamberti A., Gazia R., Bianco S., Manfredi D., Shahzad N., Cappelluti F., Ma S., Tresso E. (2012). High efficiency dye-sensitized solar cells exploiting sponge-like ZnO nanostructures. Phys. Chem. Chem. Phys..

[74] Kolodziejczak-Radzimska A., Jesionowski T. (2014). Zinc Oxide-From Synthesis to Application: A Review. Materials.

[75] Özgür Ü., Alivov Y.I., Liu C., Teke A., Reshchikov M.A., Doğan S., Avrutin V., Cho S.J., Morkoç H. (2005). A comprehensive review of ZnO materials and devices. J. Appl. Phys..

[76] Pal B., Sharon M. (2002). Enhanced photocatalytic activity of highly porous ZnO thin films prepared by sol–gel process. Mater. Chem. Phys..

[77] Rani S., Suri P., Shishodia P., Mehra R. (2008). Synthesis of nanocrystalline ZnO powder via sol–gel route for dye-sensitized solar cells. Sol. Energy Mater. Sol. Cells.

[78] Hu X., Masuda Y., Ohji T., Kato K. (2010). Fabrication of Zn(OH)2/ZnO Nanosheet-ZnO Nanoarray Hybrid Structured Films by a Dissolution-Recrystallization Route. J. Am. Ceram. Soc..

[79] Hu X., Masuda Y., Ohji T., Kato K. (2010). Dissolution−Recrystallization Induced Hierarchical Structure in ZnO: Bunched Roselike and Core−Shell-like Particles. Cryst. Growth Des..

[80] Masuda Y., Kato K. (2008). Highc-Axis Oriented Stand-Alone ZnO Self-Assembled Film. Cryst. Growth Des..

[81] Tan W.K., Razak K.A., Lockman Z., Kawamura G., Muto H., Matsuda A. (2014). Synthesis of ZnO nanorod–nanosheet composite via facile hydrothermal method and their photocatalytic activities under visible-light irradiation. J. Solid State Chem..

[82] Tan W.K., Kawamura G., Matsuda A. (2016). Design of ZnO Nano-Architectures and Its Applications.

[83] Roy P., Berger S., Schmuki P. (2011). TiO_2_ nanotubes: Synthesis and applications. Angew. Chem. Int. Ed. Engl..

[84] Tsvetkov N., Larina L., Ku Kang J., Shevaleevskiy O. (2020). Sol-Gel Processed TiO_2_ Nanotube Photoelectrodes for Dye-Sensitized Solar Cells with Enhanced Photovoltaic Performance. Nanomaterials.

[85] Thauer E., Zakharova G.S., Wegener S.A., Zhu Q., Klingeler R. (2021). Sol-gel synthesis of Li3VO4/C composites as anode materials for lithium-ion batteries. J. Alloys Compd..

[86] Tawa S., Sato Y., Orikasa Y., Matsumoto K., Hagiwara R. (2019). Lithium fluoride/iron difluoride composite prepared by a fluorolytic sol–gel method: Its electrochemical behavior and charge–discharge mechanism as a cathode material for lithium secondary batteries. J. Power Sources.

[87] Yanilmaz M., Lu Y., Zhu J., Zhang X. (2016). Silica/polyacrylonitrile hybrid nanofiber membrane separators via sol-gel and electrospinning techniques for lithium-ion batteries. J. Power Sources.

[88] Tan W.K., Asami K., Maeda Y., Hayashi K., Kawamura G., Muto H., Matsuda A. (2019). Facile formation of Fe_3_O_4_-particles decorated carbon paper and its application for all-solid-state rechargeable Fe-air battery. Appl. Surf. Sci..

[89] Tan W.K., Wada Y., Hayashi K., Kawamura G., Muto H., Matsuda A. (2019). Fabrication of an all-solid-state Zn-air battery using electroplated Zn on carbon paper and KOH-ZrO_2_ solid electrolyte. Appl. Surf. Sci..

[90] Miura A., Rosero-Navarro N.C., Sakuda A., Tadanaga K., Phuc N.H.H., Matsuda A., Machida N., Hayashi A., Tatsumisago M. (2019). Liquid-phase syntheses of sulfide electrolytes for all-solid-state lithium battery. Nat. Rev. Chem..

[91] Takano R., Tadanaga K., Hayashi A., Tatsumisago M. (2014). Low temperature synthesis of Al-doped Li7La_3_Zr_2_O_12_ solid electrolyte by a sol–gel process. Solid State Ion..

[92] Mosa J., Aparicio M. (2020). Sol-Gel Synthesis of Nanocrystalline Mesoporous Li_4_Ti_5_O_12_ Thin-Films as Anodes for Li-Ion Microbatteries. Nanomaterials.

[93] Kim S.-W., Nam K.-W., Seo D.-H., Hong J., Kim H., Gwon H., Kang K. (2012). Energy storage in composites of a redox couple host and a lithium ion host. Nano Today.

[94] Tadanaga K., Katata N., Minami T. (1997). Super-Water-Repellent Al_2_O_3_ Coating Films with High Transparency. J. Am. Ceram. Soc..

[95] Tadanaga K., Fujii T., Matsuda A., Minami T., Tatsumisago M. (2004). Micropatterning of Sol-Gel Derived Thin Films Using Hydrophobic-Hydrophilic Patterned Surface. J. Sol-Gel Sci. Technol..

[96] Nbelayim P., Sakamoto H., Kawamura G., Muto H., Matsuda A. (2017). Preparation of thermally and chemically robust superhydrophobic coating from liquid phase deposition and low voltage reversible electrowetting. Thin Solid Films.

[97] Pierre A.C. (1997). Porous sol-gel ceramics. Ceram. Int..

[98] Katagiri K., Kamiya J., Koumoto K., Inumaru K. (2012). Preparation of hollow titania and strontium titanate spheres using sol–gel derived silica gel particles as templates. J. Sol-Gel Sci. Technol..

[99] Shimogaki T., Tokoro H., Tabuchi M., Koike N., Yamashina Y., Takahashi M. (2016). Large-scale preparation of morphology-controlled microporous silica particles via gradual injection of reactants with different surfactants. J. Sol-Gel Sci. Technol..

[100] Ebrahimpour O., Dubois C., Chaouki J. (2014). Fabrication of mullite-bonded porous SiC ceramics via a sol–gel assisted in situ reaction bonding. J. Eur. Ceram. Soc..

[101] Carstens S., Enke D. (2019). Investigation of the formation process of highly porous α-Al_2_O_3_ via citric acid-assisted sol-gel synthesis. J. Eur. Ceram. Soc..

[102] Xie Y.-T., Chen J.-R., Chen Y.-T., Jiang B.-C., Sie Z.-H., Hsu H.-Y., Chen T.-L., Chiang Y.-Y., Hsueh H.-Y. (2021). Sol–gel-derived hierarchically wrinkled mesoporous ceramics for enhancement of cell alignment. Chem. Eng. J..

[103] Katagiri K., Takabatake R., Inumaru K. (2013). Robust Infrared-Shielding Coating Films Prepared Using Perhydropolysilazane and Hydrophobized Indium Tin Oxide Nanoparticles with Tuned Surface Plasmon Resonance. ACS Appl. Mater. Interfaces.

[104] Katagiri K., Narahara M., Sako K., Inumaru K. (2017). SiO_2_ shell formation mechanism and enlargement on hydrophobized nanoparticles via a reverse microemulsion process. J. Sol-Gel Sci. Technol..

[105] Chen Q., Li W., Goudouri O.M., Ding Y., Cabanas-Polo S., Boccaccini A.R. (2015). Electrophoretic deposition of antibiotic loaded PHBV microsphere-alginate composite coating with controlled delivery potential. Colloids Surf. B Biointerfaces.

[106] Muto H., Yokoi A., Tan W.K. (2020). Electrostatic Assembly Technique for Novel Composites Fabrication. J. Compos. Sci..

[107] Guzman E., Mateos-Maroto A., Ruano M., Ortega F., Rubio R.G. (2017). Layer-by-Layer polyelectrolyte assemblies for encapsulation and release of active compounds. Adv. Colloid Interface Sci..

[108] Kalogiouri N.P., Tsalbouris A., Kabir A., Furton K.G., Samanidou V.F. (2020). Synthesis and application of molecularly imprinted polymers using sol–gel matrix imprinting technology for the efficient solid-phase extraction of BPA from water. Microchem. J..

[109] Alias N., Rosli S.A., Sazalli N.A.H., Hamid H.A., Arivalakan S., Umar S.N.H., Khim B.K., Taib B.N., Keat Y.K., Razak K.A., Al-Douri Y. (2020). 15-Metal oxide for heavy metal detection and removal. Metal Oxide Powder Technologies.

[110] Shi S., Xu C., Wang X., Xie Y., Wang Y., Dong Q., Zhu L., Zhang G., Xu D. (2020). Electrospinning fabrication of flexible Fe3O4 fibers by sol-gel method with high saturation magnetization for heavy metal adsorption. Mater. Des..

[111] Mironyuk I., Mykytyn I., Vasylyeva H., Savka K. (2020). Sodium-modified mesoporous TiO_2_: Sol-gel synthesis, characterization and adsorption activity toward heavy metal cations. J. Mol. Liq..

[112] Qin H., Guo W., Huang X., Gao P., Xiao H. (2020). Preparation of yttria-stabilized ZrO_2_ nanofiltration membrane by reverse micelles-mediated sol-gel process and its application in pesticide wastewater treatment. J. Eur. Ceram. Soc..

[113] Okuno T., Kawamura G., Muto H., Matsuda A. (2015). Three modes of high-efficient photocatalysis using composites of TiO_2_-nanocrystallite-containing mesoporous SiO_2_ and Au nanoparticles. J. Sol-Gel Sci. Technol..

[114] Hill N.A. (2000). Why Are There so Few Magnetic Ferroelectrics?. J. Phys. Chem. B.

[115] Zhang L., Zhai J., Mo W., Yao X. (2010). The dielectric and leakage current behavior of CoFe_2_O_4_-BaTiO_3_ composite films prepared by combining method of sol-gel and electrophoretic deposition. Solid State Sci..

[116] Schileo G. (2013). Recent developments in ceramic multiferroic composites based on core/shell and other heterostructures obtained by sol–gel routes. Prog. Solid State Chem..

[117] Kawamura G., Ohara K., Tan W.K., Goto T., Nakamura Y., Inoue M., Muto H., Yamaguchi K., Boccaccini A.R., Matsuda A. (2018). Multiferroic nanocomposite fabrication via liquid phase using anodic alumina template. Sci. Technol. Adv. Mater..

[118] Kawamura G., Ohara K., Tan W.K., Muto H., Yamaguchi K., Boccaccini A.R., Matsuda A. (2018). Sol-gel template synthesis of BaTiO3 films with nano-periodic structures. Mater. Lett..

[119] Asadnia M., Kottapalli A.G.P., Karavitaki K.D., Warkiani M.E., Miao J., Corey D.P., Triantafyllou M. (2016). From Biological Cilia to Artificial Flow Sensors: Biomimetic Soft Polymer Nanosensors with High Sensing Performance. Sci. Rep..

[120] Kawamura G., Oura K., Tan W.K., Goto T., Nakamura Y., Yokoe D., Francis L.D., Hajraoui K.E., Wei X., Inoue M. (2019). Nanotube array-based barium titanate-cobalt ferrite composite film for affordable magnetoelectric multiferroics. J. Mater. Chem. C.

[121] Tan W.K., Oura K., Kawamura G., Boccaccini A.R., Matsuda A. (2018). Preparation of BaTiO_3_ Nanotube Arrays, CoFe_2_O_4_ Nanoparticles and Their Composites. ECS Trans..

[122] Zhang Y., Chen L., Zeng J., Zhou K., Zhang D. (2014). Aligned porous barium titanate/hydroxyapatite composites with high piezoelectric coefficients for bone tissue engineering. Mater. Sci. Eng. C.

[123] Takahashi M. (2018). Responsive and Adaptive Micro Wrinkles on Organic-inorganic Hybrid Materials. Chem. Rec..

[124] Takahashi M., Suzuki K., Tokudome Y., Malfatti L., Innocenzi P. (2014). Responsive microstructures on organic–inorganic hybrid films. J. Sol-Gel Sci. Technol..

[125] Tokudome Y., Suzuki K., Kitanaga T., Takahashi M. (2012). Hierarchical nested wrinkles on silica-polymer hybrid films: Stimuli-responsive micro periodic surface architectures. Sci. Rep..

